# From Genes to Stress Response: Genomic and Transcriptomic Data Suggest the Significance of the Inositol and Raffinose Family Oligosaccharide Pathways in *Stylosanthes scabra*, Adaptation to the Caatinga Environment

**DOI:** 10.3390/plants13131749

**Published:** 2024-06-25

**Authors:** José Ribamar Costa Ferreira-Neto, Manassés Daniel da Silva, Eliseu Binneck, Elayne Cristina Ramos Vilanova, Ana Luíza Trajano Mangueira de Melo, Jéssica Barboza da Silva, Natoniel Franklin de Melo, Valesca Pandolfi, Ana Maria Benko-Iseppon

**Affiliations:** 1 Plant Genetics and Biotechnology Laboratory, Center of Biosciences, Genetics Department, Federal University of Pernambuco, Av. Prof. Moraes Rego, 1235, Recife 50670-901, PE, Brazil; manasses.dsilva@ufpe.br (M.D.d.S.); elayne.cristina@ufpe.br (E.C.R.V.); analuiza.melo@ufpe.br (A.L.T.M.d.M.); jessica.barboza@ufpe.br (J.B.d.S.); valesca.pandolfi@ufpe.br (V.P.); 2 Embrapa Soja—Brazilian Agricultural Research Corporation (Embrapa), Rodovia Carlos João Strass–Distrito de Warta, Londrina 86085-981, PR, Brazil; eliseu.binneck@embrapa.br; 3 Embrapa Semiárido—Brazilian Agricultural Research Corporation (Embrapa), Rodovia BR-428, Km 152, s/n–Zona Rural, Petrolina 56302-970, PE, Brazil; natoniel.melo@embrapa.br

**Keywords:** raffinose family of oligosaccharides, water deprivation, drought stress, plant stress, forage legume, antinutritional factors

## Abstract

*S. scabra* is an important forage and extremophilic plant native to the Brazilian Caatinga semiarid region. It has only recently been subjected to omics-based investigations, and the generated datasets offer insights into biotechnologically significant candidates yet to be thoroughly examined. INSs (inositol and its derivatives) and RFO (raffinose oligosaccharide family) pathways emerge as pivotal candidates, given their critical roles in plant physiology. The mentioned compounds have also been linked to negative impacts on the absorption of nutrients in mammals, affecting overall nutritional intake and metabolism. Therefore, studying these metabolic pathways is important not just for plants but also for animals who depend on them as part of their diet. INS and RFO pathways in *S. scabra* stood out for their abundance of identified loci and enzymes. The enzymes exhibited genomic redundancy, being encoded by multiple loci and various gene families. The phylogenomic analysis unveiled an expansion of the PIP5K and GolS gene families relative to the immediate *S. scabra* ancestor. These enzymes are crucial for synthesizing key secondary messengers and the RFO precursor, respectively. Transcriptional control of the studied pathways was associated with DOF-type, C_2_H_2_, and BCP1 transcription factors. Identification of biological processes related to INS and RFO metabolic routes in *S. scabra* highlighted their significance in responding to stressful conditions prevalent in the Caatinga environment. Finally, RNA-Seq and qPCR data revealed the relevant influence of genes of the INS and RFO pathways in the *S. scabra* response to water deprivation. Our study deciphers the genetics and transcriptomics of the INS and RFO in *S. scabra*, shedding light on their importance for a Caatinga-native plant and paving the way for future biotechnological applications in this species and beyond.

## 1. Introduction

*Stylosanthes scabra*, a forage legume indigenous to the Caatinga environment, dominates much of Brazil’s semi-arid region. The flora within the referred geographical realm is renowned for its resilience, adapting remarkably to harsh environmental conditions characterized by high temperatures, saline soils, and water scarcity [1].

Notably, the mentioned plant demonstrates rapid perception and response to fluctuations in soil moisture [2] and possesses substantial osmotic adjustment capabilities, crucial for maintaining tissue turgor under low water potential conditions [3]. This ability serves as a critical survival strategy in semiarid environments. Additionally, its extensive root system enables efficient water uptake, even under extremely low soil moisture conditions, often below −1.5 MPa, a threshold typically associated with the wilting point.

Despite its physiological and adaptive resilience, *S. scabra* has only recently been subjected to omics-based investigations. Our research endeavors have delved into transcriptomics [2] and genomics [4] to unravel their molecular intricacies and elucidate their response mechanisms to abiotic stressors. Transcriptomic and physiological analyses of the *S. scabra* root tissue under water deprivation unveiled the prominence of molecular players involved in reactive oxygen species (ROS) scavenging, amino acid biosynthesis, and response to abscisic acid [2]. Furthermore, aquaporins from the TIP subfamily exhibited significant up-regulation under the studied stress condition [4]. On the genomic front, *S. scabra* gene families associated with ROS scavenging, defense system modulation, secondary compound biosynthesis (e.g., proline), and cell wall dynamics were found to be genomically expanded compared to its immediate ancestor [4]. Collectively, these findings unveil a critical arsenal of molecular players potentially employed by the species to adapt to its harsh habitat.

The mentioned omics datasets serve as invaluable reservoirs of information, offering insights into biotechnologically significant candidates yet to be thoroughly examined. Among the myriad possibilities, the exploitation of the inositol metabolic pathway and its derivatives (INSs) emerges as particularly promising. Inositol molecules possess the capacity for methylation (yielding pinitol) or phosphorylation (yielding phosphoinositides, polyphosphoinositides, inositol phosphates, or inositol polyphosphates). Additionally, myo-inositol plays a crucial role in the metabolism of the raffinose family of oligosaccharides (RFOs), serving as a pivotal precursor in their biosynthetic pathway [5].

The compounds mentioned above play key roles in plants. Methylated derivatives of inositol have been linked to adaptive responses under drought stress [6,7]. Conversely, the phosphorylated ones are regarded as crucial secondary messengers. The signal transduction amplification generated by these molecules positions them as “master regulators”, modulating essential molecular components involved in response to osmotic stressors [8,9], which are commonly encountered in the Caatinga environment. Concerning RFO, these compounds are implicated in abiotic stress tolerance mechanisms across various plant species [10,11]. For mammals, however, certain types of INS and RFO are considered antinutritional factors [12,13]. These substances have been associated with adverse effects on the absorption of nutrients, thereby impacting overall nutritional intake and metabolism. Hence, the investigation of these metabolic routes holds implications not only for the plants themselves but also for those who rely on them as part of their diet.

In the realm of legumes, our research group has underscored the significant involvement of INS and RFO in responding to root dehydration stresses. Omics analyses have shed light on soybean [14] and cowpea [15] plants. In this context, the present study aims to capitalize on the recently generated *S. scabra* genomic and transcriptomic resources to deepen our understanding of these metabolic pathways in legumes. Since certain INS molecules constitute crucial secondary messengers, the transcriptional orchestration of their metabolic pathway, alongside that of RFO, was scrutinized following 24 h of water deprivation. The chosen treatment duration aimed to capture the initial response moments to the studied scenario. Upon early detection of specific biotic or abiotic stressors, plants initiate appropriate actions that could lead to successful acclimatization. Furthermore, we conducted loci mining of enzymes associated with the mentioned pathways in *S. scabra*, analyzed the genomic expansion (or contraction) of its potential gene families, determined potential biological processes in which the pathways are involved, and identified transcription factors regulating INS and RFO syntheses in the species under investigation. Through these comprehensive analyses, we aim to contribute to a more nuanced understanding of the mentioned pathways in plants, potentially paving the way for enhanced resilience in the face of environmental challenges.

## 2. Material and Methods

### 2.1. Maps for INS and RFO Metabolic Pathways

The metabolic map for INS can be accessed on the “Kyoto Encyclopedia of Genes and Genomes” (KEGG) database (http://www.genome.jp/kegg/pathway.html (accessed on 15 December 2023)) under “Inositol Phosphate Metabolism” (map00562). Similarly, the corresponding map for RFO is available as a subset of reactions within the “Galactose Metabolism map” (map00052). Although the KEGG Pathway database lacks specific information on these pathways for *S. scabra*, we have leveraged data from *Arachis hypogaea* as a reference. *A. hypogaea* serves as the closest phylogenetic relative to *S. scabra* [4] with available genomic data in the KEGG database. Thus, we utilized their INS and RFO metabolic pathway information as a proxy to infer potential similarities and glean insights into the studied organism.

### 2.2. Genomic Approach

#### 2.2.1. *S. scabra* Genomic Resource

The *S. scabra* genomic sequences utilized in the present study are sourced from the investigation conducted by Ferreira-Neto et al. [4]. The genomic sequences that underpin the findings of this research are accessible in the NCBI Bioproject repository (https://www.ncbi.nlm.nih.gov/bioproject/) under the accession PRJNA924790.

#### 2.2.2. INS and RFO Gene/Transcripts Mining

The protein sequences of INS and RFO enzymes, previously identified in *A. hypogaea* in the KEGG database and available in the NCBI Protein database, served as “seed” sequences for homologous search. To identify corresponding sequences in the organism under scrutiny, the “seed” sequences were aligned using BLASTp against the *S. scabra* conceptual proteome and translated RNA-Seq data. The alignment parameters were set as follows:

The e-value < 10^−10^: this parameter ensures the statistical significance of the alignments, allowing for the identification of homologous sequences with a low chance of random matches.

Alignment of at least 200 amino acids: this criterion ensures that the aligned regions are sufficiently long to identify homologous proteins confidently.

The qcovs (Query Coverage Per Subject) ≥ 80%: this parameter ensures that the aligned regions cover at least 80% of the query sequence, indicating a significant match.

Furthermore, we conducted a manual verification to confirm the annotations and ensure the accuracy of the identified sequences.

#### 2.2.3. Orthogroups Identification and Respective Expansion/Contraction Analysis

As described by Emms and Kelly [16], an orthogroup refers to the collection of genes originating from a single gene found in the last common ancestor (LCA) of a specific group of species. Essentially, an orthogroup represents the natural expansion of orthology, encompassing gene sets across multiple species. In our quest to identify orthogroups, conceptual proteome data containing primary proteins were obtained from eleven distinct species, including *S. scabra*, eight Fabaceae species (*Vigna unguiculata*, *Phaseolus vulgaris*, *Glycine max*, *Cicer arietinum*, *Cajanus cajan*, *Lotus japonicus*, *Arachys hypogaea*, and *Trifolium pratense*), one Solanaceae species (*Manihot esculenta*); one Salicaceae species (*Populus trichocarpa*), and one Brassicaceae species (*Arabidopsis thaliana*). Following data retrieval, a similarity analysis was conducted using the DIAMOND tool [17], with a specified e-value threshold of less than 10^−5^. Subsequently, the obtained data were organized into orthogroups, also known as gene families, utilizing the Orthofinder software (version 2.4.0) pipeline.

To evaluate the expansion and contraction of the *S. scabra* orthogroups, we conducted an analysis using the CAFE5 software (version 5) [18], applying default parameters. For estimating the divergence time between *S. scabra* and *A. thaliana* (the selected outgroup), we obtained a dated species tree from the TimeTree database [19], which served as a guide tree. The species tree was inferred through STAG (https://github.com/davidemms/STAG and rooted with STRIDE (https://github.com/davidemms/STRIDE, both of which are integrated into the Orthofinder tool. The café5 lambda parameter, representing the birth-death rate, was estimated using gene families, wherein no more than 100 genes originated from a single genome. Orthogroups demonstrating a significant rate of expansion or contraction were identified using a threshold conditional *p*-value (*p* < 0.05). From this subset of data, we filtered identifiers associated with enzymes of the INS and RFO metabolic pathways, along with their corresponding orthogroups.

#### 2.2.4. Cis-Regulatory Elements Mining and Respective Transcription Factors Identification

The promoter regions (1.0 kb range) of the INS and RFO genes were extracted from the *S. scabra* genome using the Bedtools toolset (https://bedtools.readthedocs.io/en/latest/ (accessed on 10 January 2024)). Subsequently, the MEME v5.0.3 software was utilized to identify motifs, also referred to as candidate cis-regulatory elements (CCREs), within each promoter region. For characterization, an e-value threshold of e < 10^−2^ was applied, classifying motifs with e-values below this threshold as enriched or bona fide CCRE. The analysis considered a maximum of 10 motifs per promoter region, with motif lengths ranging from 6 to 50 nucleotides.

To associate transcription factors (TFs) with the identified bona fide CCRE, the TomTom software v4.11.2 was employed in conjunction with the JASPAR database (Jaspar_Core_2022_Plants). This process entailed aligning the enriched bona fide CCRE obtained from MEME with the annotated motifs in the JASPAR database. Statistical criteria, including a *p*-value cutoff of <10^−2^ and a false discovery rate (FDR) cutoff of <10^−2^, were applied to identify valid alignments.

#### 2.2.5. GO Enrichment Analysis

To investigate the enriched biological processes associated with INS and RFO genes, gene ontology enrichment analysis was conducted using the PlantRegMap tool [20]. The Singular Enrichment Analysis (*p* < 0.05) method was employed for this purpose. The results of the referred test were then summarized and visualized using the REVIGO tool [21]. The background data used for the analysis consisted of the conceptual *S. scabra* proteome, which was annotated through BLASTp with a specified e-value threshold of less than 10^−10^. The BLASTp annotation was performed against sequences from *A. hypogaea*, *Arachis ipaensis*, and *Arachis duranensis*, all of which were available in the Uniprot database (https://www.uniprot.org/ (accessed on 20 January 2024)).

### 2.3. Transcriptomic Approach

#### 2.3.1. *S. scabra* Transcriptomic Resource

The *S. scabra* gene expression data utilized in the present study are sourced from the investigation conducted by Ferreira-Neto et al. [2]. The RNA-Seq libraries that underpin the findings of this research are accessible in the NCBI SRA (https://www.ncbi.nlm.nih.gov/sra repository under the accession PRJNA837909. The following items provide a summary of the conditions under which the aforementioned libraries were obtained.

#### 2.3.2. Plant Material, Growing Conditions, and Stress Application

The used *S. scabra* accession (85/UNEB) was collected in the Caatinga environment and is part of the Active Germplasm Bank of the Universidade do Estado da Bahia (UNEB)—Brazil. Detailed information regarding the growth and cultivation conditions can be found in Ferreira-Neto et al. [2]. In summary, plant propagation involved the use of stem cuttings measuring 10 cm in length at EMBRAPA SEMIÁRIDO (Petrolina, Pernambuco), Brazil. These cuttings were then placed in a climate-controlled greenhouse with 50% shade and received irrigation twice a day. Once the plants developed roots, they were transferred to plastic pots (10 L) filled with a substrate consisting of sand–ultisol-vermiculite. In this stage, the plants were watered once daily, specifically at 9 a.m.

After six months of cultivation, the plants underwent water deprivation through the suspension of irrigation for a period of 24 h (WD.24h treatment). To impose the water deprivation condition, the treatment plants were not watered at the usual 9 a.m time. After 24 h, we collected root tissue from these plants (WD.24h treatment) for analysis. There was also a corresponding irrigated control group (Cont.24h treatment). To ensure the accuracy of the results, all experiments were conducted in triplicate, with each biological replicate consisting of three plants per pot. Following collection, the plants were immediately frozen using liquid nitrogen, while the roots were stored separately in an ultra-freezer at −80 °C.

#### 2.3.3. RNA-Seq Libraries: Synthesis and Sequencing

Total RNA was isolated from the roots of treated and control plants using the “SV Total RNA Isolation System” kit (Promega, Madison, WI, USA) following the manufacturer’s protocol. The concentration, purity, and integrity of the extracted RNA were assessed using a Qubit Fluorometer (Thermo Fisher Scientific, Waltham, MA, USA), NanoDrop^®^ 2000 (Thermo Fisher Scientific), and the Agilent 2100 Bioanalyzer (Agilent Technologies, Santa Clara, CA, USA), respectively. Only samples with an RNA integrity number (RIN) of ≥8.0 were selected for sequencing. The same sequencing platform and kits used in the study by Ferreira-Neto et al. [2] were employed in the current research. For the Cont.24h/WD.24h treatments, a total of six RNA-Seq libraries were synthesized, encompassing all the biological replicates.

#### 2.3.4. RNA-Seq Library Assembly, Transcriptome Annotation, and Differential Expression Analysis

The comprehensive methodology employed in this phase, along with the metrics of the resultant assembly, is detailed in Ferreira-Neto et al. [2]. Here, we provide a concise summary of the procedural steps undertaken. Initially, adapters and low-quality sequences were eliminated using the Trimmomatic software (version 0.39) [22]. Subsequently, the remaining reads exhibited a Phred value of ≥30. Quality assessments of the resultant sequences were conducted using FastQC 0.11.8 software (https://github.com/s-andrews/FastQC (accessed on 10 January 2022)).

For data assembly and subsequent analysis, the “de novo pipeline RNA-Seq” (version 3.1.3) from the GenPipes platform [23] was utilized. The transcriptome assembly was conducted employing Trinity 2.0.4 software [24]. Following assembly, Transdecoder 2.0.1 (https://github.com/TransDecoder/TransDecoder/wiki (accessed on 10 January 2022)) was employed to identify and translate candidate ORFs, with preference given to the largest ORF within each transcript. Subsequently, functional annotation of the identified ORFs in the resulting transcriptomes was accomplished using Trinotate 2.0.2 (https://github.com/Trinotate/Trinotate.github.io/wiki (accessed on 10 January 2022)). Completeness assessment of the transcriptome assembly was executed utilizing gVolante [25] and the ortholog search pipeline “BUSCO” v.5 (ortholog set: Viridiplantae). For the differential expression analysis, the RNA-Seq transcripts underwent edgeR analysis [26]. In this procedure, transcripts exhibiting log2 fold change (Log2FC) values equal to or less than “−2” and greater than or equal to “2” associated with *p*-values below 0.05 and false discovery rate (FDR) below 0.05 were considered, respectively, down-regulated (DR) and up-regulated (UR). Transcripts falling outside of the specified statistics were considered constitutively expressed.

#### 2.3.5. Metabolic Pathway Analyses Using the “KEGG Mapper-Search & Color Pathway” Tool

To visually analyze the transcriptional orchestration of the INS and RFO pathways, we integrated RNA-Seq expression data with the KEGG Mapper-Search & Color Pathway tool. Transcriptional modulations were depicted using color codes on nodes containing the EC (Enzyme Commission) numbers as follows: Red for enzymes strictly UR; Green for strictly DR enzymes; Black for those with constitutive expression; and Orange for highlight enzymes with both UR and DR transcripts.

In this manuscript, enzymes with UR and DR transcriptional isoforms (i.e., orange highlighted in the map) were interpreted as UR. This classification implies that at least one transcript (or a specific group of transcripts) encoding a given enzyme was responsive to the conditions under investigation.

#### 2.3.6. RNA-Seq Data Validation

The qPCR analyses of INS and RFO transcripts (with a focus on those up-regulated in the RNA-Seq libraries) were conducted following the MIQE guidelines [27] to ensure robust and reproducible results. To ensure statistical reliability, three biological replicates and three technical replicates were employed in the reactions. The SYBR Green detection method was used, and the reactions were performed in 96-well plates using the LineGene 9660 system (Bioer, Hangzhou, China). The same total RNA samples used for RNA-Seq library sequencing were utilized in the qPCR analysis. RNA quantity was assessed using a Qubit fluorometer, and quality screening was performed using agarose gel electrophoresis and the NanoDrop^®^ 2000 spectrophotometer before cDNA synthesis. The cDNA synthesis protocol followed the guidelines provided by GoScript™ Reverse Transcriptase (Promega). The primer pairs design, reference genes used, primer pairs efficiency evaluation (Supplementary Material S1), and melting curve analysis (Supplementary Material S2) were conducted in accordance with Ferreira-Neto et al. [4].

To determine the relative expression of INS and RFO target transcripts, the Rest2009 software (version REST-2008; standard mode) was utilized. This analysis relies on paired comparisons between the target transcripts and reference genes under both stress and control conditions. The method employs randomization and bootstrapping—specifically, the Pair-wise Fixed Reallocation Randomization Test© [28]—to determine the statistical significance of the differences in expression between the target transcripts under control and treated conditions. Hypothesis testing (*p* < 0.05) was employed to assess whether the differences in expression of the target transcripts between control and treated conditions were statistically significant.

### 2.4. Artificial Intelligence Use

This work utilized generative artificial intelligence (AI) to facilitate the initial translation (Portuguese to American English) of the present manuscript. The AI tool (ChatGPT; https://chat.openai.com/ (accessed on 20 March 2024)) provided a preliminary translation, which was subsequently reviewed, edited, and verified by human experts for accuracy and scientific fidelity. This approach aimed to expedite the comprehension of the original source material while maintaining the rigor and integrity of the scientific content presented.

## 3. Results

### 3.1. INS and RFO Pathways in S. scabra

**Figure 1:**
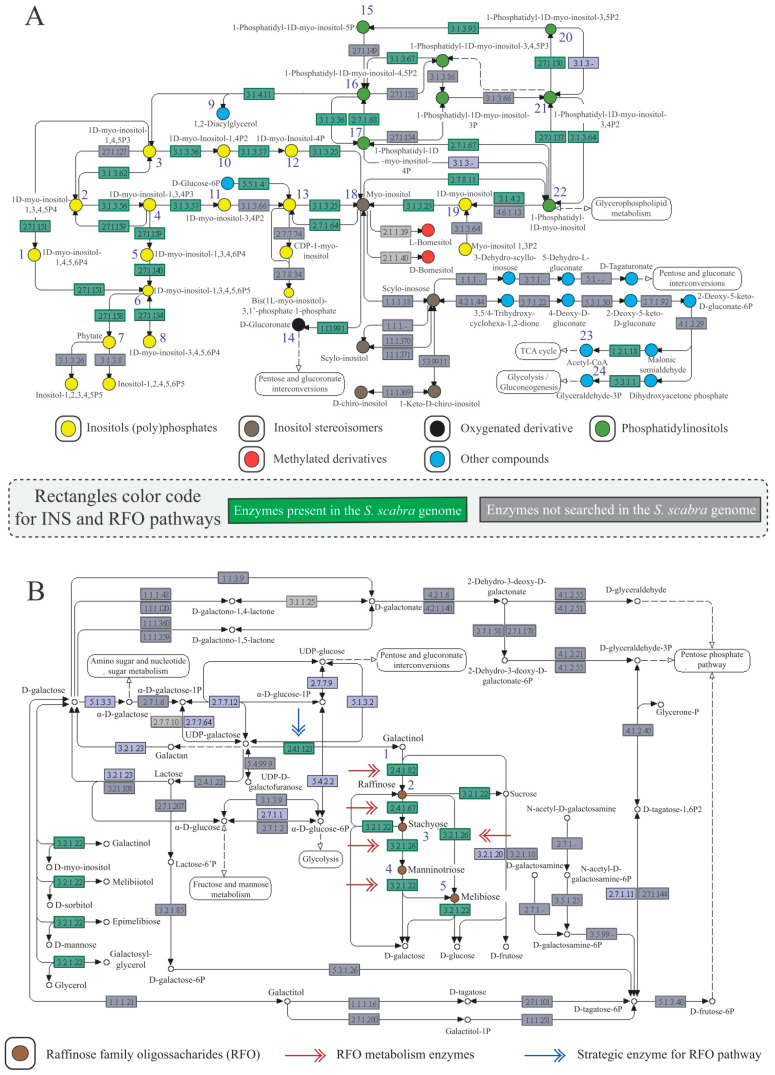
Metabolic maps for INS (**A**) and RFO (**B**) pathways extracted from the KEGG Pathway database. Legend: Numbers in blue indicate the total count of compounds for which synthesizing enzymes were identified in the *Stylosanthes scabra* genome. Green rectangles denote enzymes with genomic information sourced from the reference organism (*Arachis hypogaea*) and exhibiting homologs in the *S. scabra* genome. Conversely, gray nodes represent enzymes lacking genomic data for the reference species *A. hypogaea*, thus not searched in the *S. scabra* genome; INS (inositol and derivatives); RFO (raffinose oligosaccharides family); INS Pathway [EC 1.13.99.1: myo-inositol oxygenase; EC 1.2.1.27|1.2.1.18: methylmalonate-semialdehyde dehydrogenase; EC 2.7.1.134|2.7.1.159: inositol-tetrakisphosphate 1-kinase 3; EC 2.7.1.137: phosphatidylinositol 3-kinase; EC 2.7.1.150: 1-phosphatidylinositol-3-phosphate 5-kinase; EC 2.7.1.151|2.7.1.140: inositol polyphosphate multikinase; EC 2.7.1.158: inositol-pentakisphosphate 2-kinase; EC 2.7.1.64: myo-inositol 3-kinase; EC 2.7.1.67: phosphatidylinositol 4-kinase; EC 2.7.1.68: phosphatidylinositol 4-phosphate 5-kinase 4-like; EC 2.7.8.11: phosphatidylinositol synthase; EC 3.1.3.25|3.1.3.93|3.1.3.15: inositol-phosphate phosphatase; EC 3.1.3.36: inositol polyphosphate 5-phosphatase 9; EC:3.1.3.56: type I inositol polyphosphate 5-phosphatase IP5P1/2; EC 3.1.3.57|3.1.3.7: SAL1 (3′(2′),5′-bisphosphate nucleotidase 1); EC 3.1.3.64|3.1.3.95: myotubularin-related protein 1/2; EC 3.1.3.67|3.1.3.48|3.1.3.16: PTEN; EC 3.1.3.80|3.1.3.62: multiple inositol polyphosphate phosphatase 1; EC 3.1.4.11: phosphoinositide phospholipase C; EC 3.1.4.3: non-specific phospholipase C; EC 5.3.1.1: triosephosphate isomerase; EC 5.5.1.4: myo-inositol-3-phosphate synthase]; RFO Pathway [EC 2.4.1.123: galactinol synthase; EC 2.4.1.82: raffinose synthase; EC 2.4.1.67: stachyose synthase; EC 3.2.1.26: invertase (or beta-fructofuranosidase); EC 3.2.1.22: α-galactosidase].

The current model of the INS metabolic pathway comprises 44 different enzymes contained in 61 nodes (rectangles in Figure 1A). Genomic information for 22 enzymes (green rectangles in Figure 1A) was obtained using *A. hypogaea* as a reference. That group of enzymes facilitates the synthesis of 24 different compounds (blue numbers in Figure 1A), categorized into five types of INSs (Figure 1A): inositol phosphates, inositol stereoisomers, oxygenated derivatives, phosphatidylinositols, and others. All 22 mentioned enzymes have coding loci in the *S. scabra* genome, suggesting that this species has the capacity to synthesize a wide range of INS compounds. Enzymes within gray rectangles (Figure 1A), including those responsible for synthesizing methylated inositols and some inositol stereoisomers, were not identified in the genomic data of the reference species (*A. hypogaea*).

The galactose metabolic pathway contains 48 enzymes distributed across 61 nodes (Figure 1B). Within this pathway, the RFO map constitutes a specific subset, featuring five enzymes (indicated by blue and red arrows associated with green rectangles). Among these enzymes, galactinol synthase (EC:2.4.1.123) plays a crucial role in synthesizing the RFO precursor, denominated galactinol (Figure 1B). The remaining four enzymes, namely raffinose synthase (EC:2.4.1.82), stachyose synthetase (EC:2.4.1.67), beta-fructofuranosidase (EC:3.2.1.26), and alpha-galactosidase (EC:3.2.1.22), are responsible for synthesizing the actual RFO compounds: raffinose, stachyose, mannitriose, and melibiose, respectively (Figure 1B). All the mentioned enzymes presented coding loci in the *S. scabra* genome.

### 3.2. Gene Mining and Phylogenomic Dynamics of INS and RFO Synthesizing Gene Families

**Figure 2:**
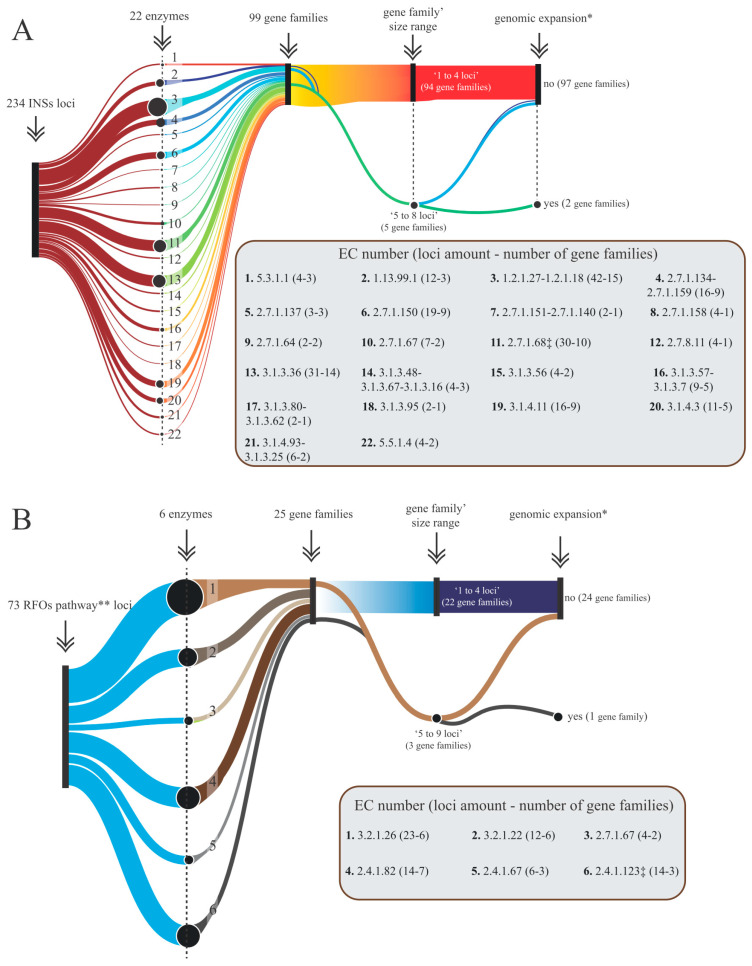
Sankey diagram illustrating genomic information of INS (**A**) and RFO (**B**) pathways, depicting the total number of identified loci for each one, the number of analyzed enzymes, their corresponding numbers of loci and gene families, and phylogenomic analysis of expansion or contraction compared to the immediate ancestor of *S. scabra*. Legend: INS (inositol and derivatives); RFO (raffinose oligosaccharides family); INS Pathway [EC 1.13.99.1: myo-inositol oxygenase; EC 1.2.1.27|1.2.1.18: methylmalonate-semialdehyde dehydrogenase; EC 2.7.1.134|2.7.1.159: inositol-tetrakisphosphate 1-kinase 3; EC 2.7.1.137: phosphatidylinositol 3-kinase; EC 2.7.1.150: 1-phosphatidylinositol-3-phosphate 5-kinase; EC 2.7.1.151|2.7.1.140: inositol polyphosphate multikinase; EC 2.7.1.158: inositol-pentakisphosphate 2-kinase; EC 2.7.1.64: myo-inositol 3-kinase; EC 2.7.1.67: phosphatidylinositol 4-kinase; EC 2.7.1.68: phosphatidylinositol 4-phosphate 5-kinase 4-like; EC 2.7.8.11: phosphatidylinositol synthase; EC 3.1.3.25|3.1.3.93|3.1.3.15: inositol-phosphate phosphatase; EC 3.1.3.36: inositol polyphosphate 5-phosphatase 9; EC:3.1.3.56: type I inositol polyphosphate 5-phosphatase IP5P1/2; EC 3.1.3.57|3.1.3.7: SAL1 (3′(2′),5′-bisphosphate nucleotidase 1); EC 3.1.3.64|3.1.3.95: myotubularin-related protein 1/2; EC 3.1.3.67|3.1.3.48|3.1.3.16: PTEN; EC 3.1.3.80|3.1.3.62: multiple inositol polyphosphate phosphatase 1; EC 3.1.4.11: phosphoinositide phospholipase C; EC 3.1.4.3: non-specific phospholipase C; EC 5.3.1.1: triosephosphate isomerase; EC 5.5.1.4: myo-inositol-3-phosphate synthase]; RFO Pathway [EC 2.4.1.123: galactinol synthase; EC 2.4.1.82: raffinose synthase; EC 2.4.1.67: stachyose synthase; EC 3.2.1.26: invertase (or beta-fructofuranosidase); EC 3.2.1.22: α-galactosidase]; * *p* < 0.05; ** Galactinol synthase and RFO-synthesizing enzymes.

The 22 INS-analyzed enzymes were encoded by 234 distinct loci. Each enzyme was encoded by more than one locus, with the number ranging from two (e.g., myo-inositol 3-kinase, EC 2.7.1.64; Figure 2A) to forty-two (e.g., methylmalonate-semialdehyde dehydrogenase, EC 1.2.1.27|1.2.1.18; Figure 2A). Similarly, for the RFO pathway, encoded by 73 loci, genomic redundancy was observed for all constituent enzymes, with the number of loci ranging from four (e.g., stachyose synthase, EC 2.4.1.67; Figure 2B) to twenty-three (e.g., invertase, EC 3.2.1.26; Figure 2B).

In addition to the observed genomic redundancy, it was noted that most enzymes in the INS and RFO pathways were encoded by multiple gene families (GFs) or orthogroups (Figure 2A,B). Seventeen (77%) of the twenty-two INS enzymes were encoded by 2 to 16 different GF, and all RFO enzymes were encoded by more than 1 GF (Figure 2A,B). Notably, the majority of GF in both pathways consisted of one to four genes (Figure 2A,B).

In terms of the phylogenomic dynamics of GF expansion or contraction, it was observed that the majority of them did not undergo significant expansion or contraction compared to the immediate ancestor of *S. scabra* (Figure 2A,B). However, phosphatidylinositol 4-phosphate 5-kinase 4-like (EC 2.7.1.68), associated with the INS pathway, demonstrated a notable expansion (*p* < 0.05) of two GFs, while galactinol synthase (EC 2.4.1.123), related to the RFO pathway, exhibited expansion (*p* < 0.05) of one GF (Figure 2A,B).

### 3.3. Bona Fide CCRE and Associated Transcription Factors

**Figure 3:**
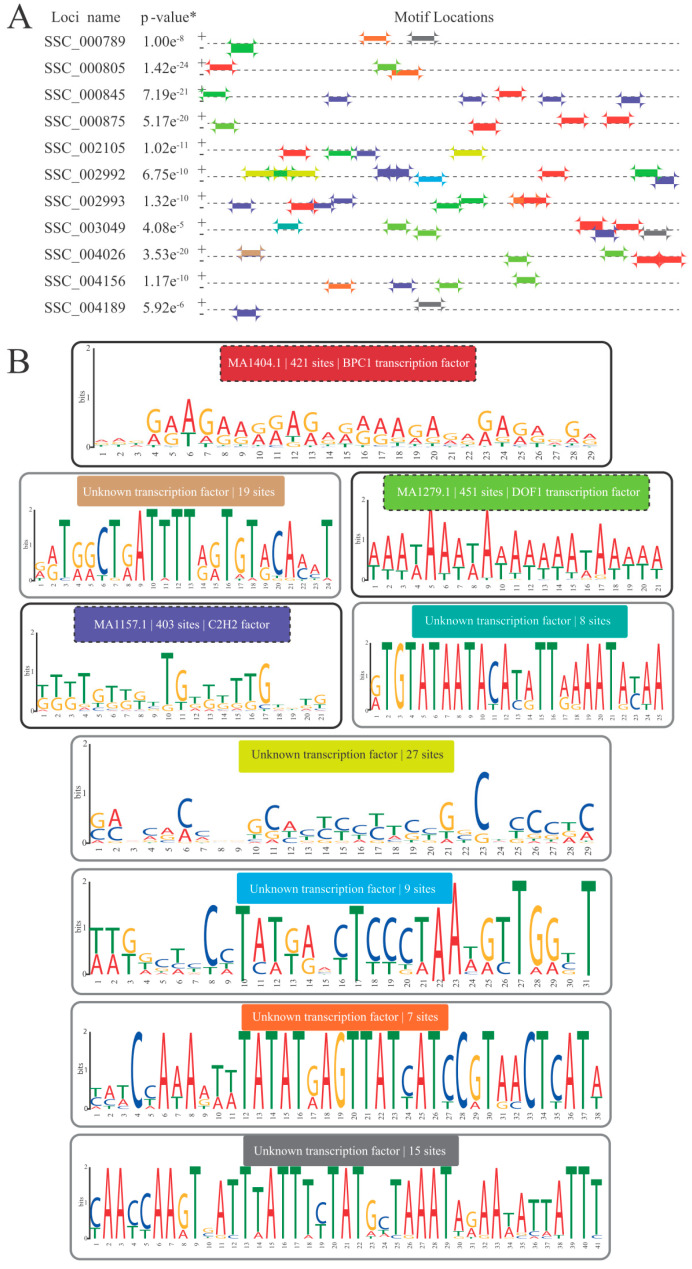
Content and distribution of bona fide CCRE anchored in promoter regions of genes from the INS and RFO pathways. (**A**) Sample of genes encoding enzymes from the INS and RFO pathways with the distribution (colored rectangles) of identified bona fide CCRE. (**B**) Presentation of the logo sequence of the identified bona fide CCRE, highlighting the associated transcription factors, their respective JASPAR Matrix IDs, and the number of anchoring sites (information inside the colored rectangles). Legend: the symbols “+” and “−” represent the sense and antisense strands of the promoter regions analyzed. * This is the combined MEME match *p*-value, defined as the probability that a random sequence (with the same length and conforming to the background) would have position *p*-values such that the product is smaller or equal to the value calculated for the tested sequence. All motifs demonstrated statistical significance below the adopted cutoff (e-value < 10^−2^).

The promoter regions (1 kb range) of the 307 genes (234 for INS and 73 for RFO; Figure 3), constituting the analyzed pathways, were examined to identify motifs corresponding to CCRE and their associated transcription factors. Nine different motifs, distributed across 1360 sites along the promoters of INS and RFO genes, were identified as enriched and considered bona fide CCRE (see Section 2.2.4). Three of these motifs (colored rectangles with a dashed border; Figure 3), accounting for approximately 93% of the total, exhibiting an average of at least one motif per investigated promoter. This subset of motifs was linked to transcription factors with identified identities, namely (Figure 3) BCP1 (421 sites), DOF-type (451 sites), and C_2_H_2_ (403 sites).

### 3.4. Enriched Biological Processes for the INS and RFO Pathways in S. scabra

**Figure 4:**
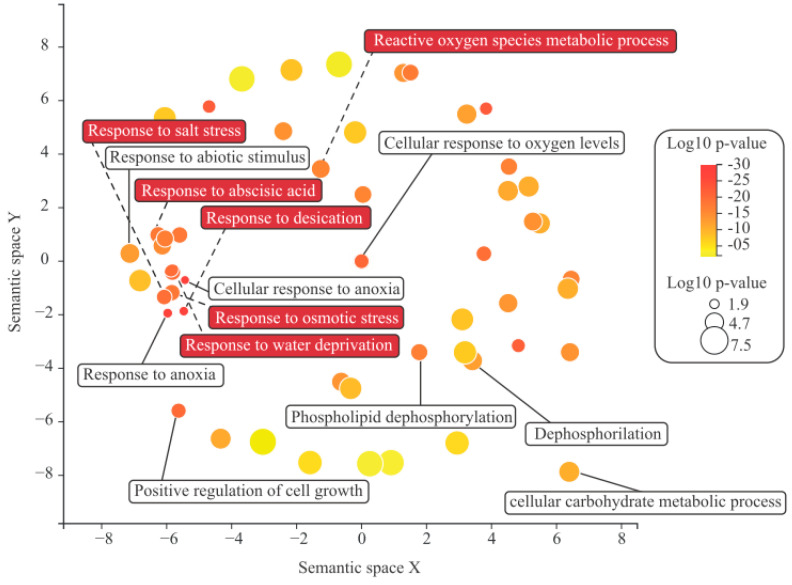
Sample of enriched biological processes associated with genes encoding enzymes of the INS and RFO pathways in *S. scabra*. Legend: terms related to dehydration stress response are highlighted in red. The color of each bubble corresponds to the *p*-value, with the referential located in the top right corner. Additionally, the “Log size” indicates the frequency of the Gene Ontology (GO) term in the background data utilized, where bubbles representing more general terms appear larger.

The studied genes were collectively analyzed to identify potentially enriched biological processes that could contribute to understanding their possible impacts on the molecular physiology of *S. scabra*. A total of 93 enriched terms were identified. A significant portion of this set represents terms associated with generic actions (such as “alcohol biosynthetic process”, “single-organism carbohydrate metabolic process”, etc.). However, a subset of these terms proved informative, being associated with highly specific terms mainly related to responses to dehydration stress (Figure 4), including: “response to saline stress”, “desiccation response”, “osmotic stress response”, “abscisic acid response”, and “water deprivation response”. Additionally, the term “reactive oxygen species metabolic process” (Figure 4), commonly associated with stress responses in general, was also identified.

### 3.5. Transcriptomics of the INS and RFO Pathways under WD.24h Treatment

**Figure 5:**
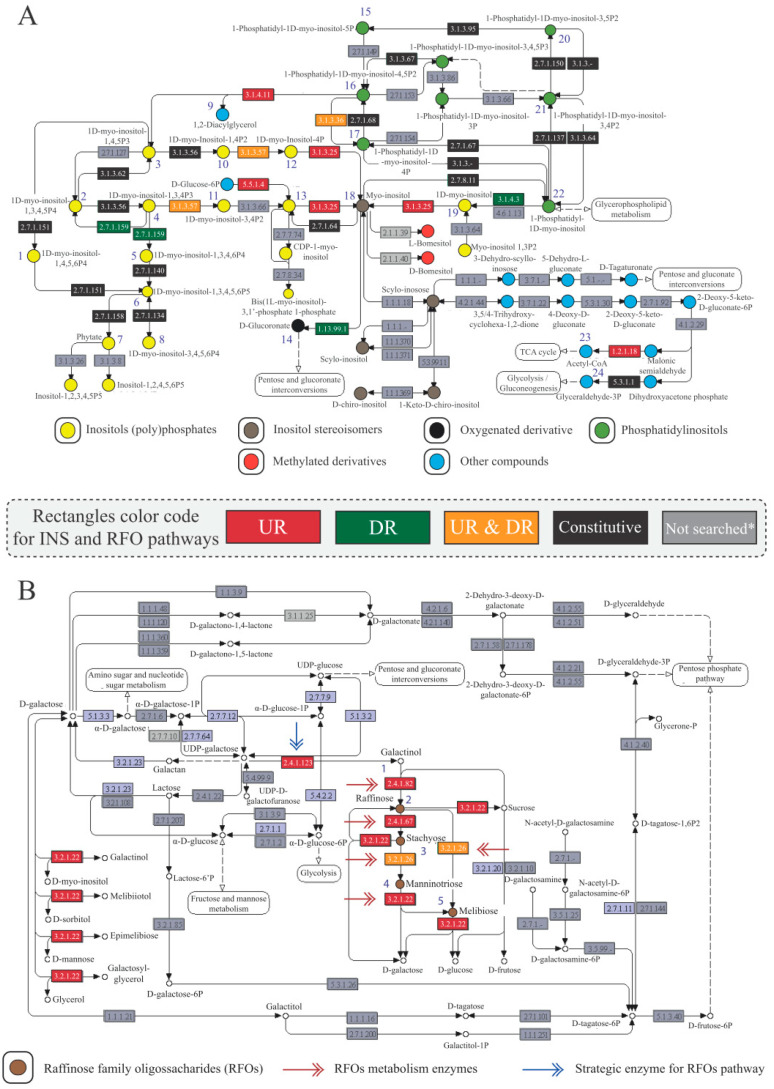
Transcriptional orchestration of the INS (**A**) and RFO (**B**) metabolic pathways in *S. scabra* under WD.24h treatment. Legend: WD.24h treatment (24 h water withholding); blue numbers indicate the total number of compounds whose synthesizing enzymes were identified in the analyzed genome; * denotes enzymes without genomic information available for the species *A. hypogea* (used as a reference) and, therefore, not searched in the *S. scabra* genome; INS Pathway [EC 1.13.99.1: myo-inositol oxygenase; EC 1.2.1.27|1.2.1.18: methylmalonate-semialdehyde dehydrogenase; EC 2.7.1.134|2.7.1.159: inositol-tetrakisphosphate 1-kinase 3; EC 2.7.1.137: phosphatidylinositol 3-kinase; EC 2.7.1.150: 1-phosphatidylinositol-3-phosphate 5-kinase; EC 2.7.1.151|2.7.1.140: inositol polyphosphate multikinase; EC 2.7.1.158: inositol-pentakisphosphate 2-kinase; EC 2.7.1.64: myo-inositol 3-kinase; EC 2.7.1.67: phosphatidylinositol 4-kinase; EC 2.7.1.68: phosphatidylinositol 4-phosphate 5-kinase 4-like; EC 2.7.8.11: phosphatidylinositol synthase; EC 3.1.3.25|3.1.3.93|3.1.3.15: inositol-phosphate phosphatase; EC 3.1.3.36: inositol polyphosphate 5-phosphatase 9; EC:3.1.3.56: type I inositol polyphosphate 5-phosphatase IP5P1/2; EC 3.1.3.57|3.1.3.7: SAL1 (3′(2′),5′-bisphosphate nucleotidase 1); EC 3.1.3.64|3.1.3.95: myotubularin-related protein 1/2; EC 3.1.3.67|3.1.3.48|3.1.3.16: PTEN; EC 3.1.3.80|3.1.3.62: multiple inositol polyphosphate phosphatase 1; EC 3.1.4.11: phosphoinositide phospholipase C; EC 3.1.4.3: non-specific phospholipase C; EC 5.3.1.1: triosephosphate isomerase; EC 5.5.1.4: myo-inositol-3-phosphate synthase]; RFO Pathway [EC 2.4.1.123: galactinol synthase; EC 2.4.1.82: raffinose synthase; EC 2.4.1.67: stachyose synthase; EC 3.2.1.26: invertase (ou beta-fructofuranosidase); EC 3.2.1.22: α-galactosidase].

For the WD.24h treatment, the analysis of transcriptional orchestration of the INS and RFO pathways through RNA-Seq data revealed how different scrutinized enzymes respond to the studied stress. In the INS pathway, DR was observed in only three enzymes, constituting 13% of the total analyzed pathway. These enzymes include inositol-tetrakisphosphate 1-kinase 3 (EC 2.7.1.134|2.7.1.159), myo-inositol oxygenase (EC 1.13.99.1), and non-specific phospholipase C (EC 3.1.4.3), as indicated by green rectangles in Figure 5A. The remaining 87% of the enzymes showed strictly constitutive expression (black rectangles in Figure 5A), or at least one of their coding isoforms showed UR (red or orange rectangles in Figure 5A). This observation suggests that a significant portion of the INS pathway does not exhibit transcriptional bottlenecks, with the majority of enzymes maintaining consistent or increased levels of associated transcripts.

Among the enzymes with constitutive expression, the majority are involved in the synthesis of inositol polyphosphates, phosphatidylinositols, and other compounds (which serve as entry points for different metabolic pathways) (Figure 5A). On the other hand, the enzymatic group exhibiting UR isoforms includes phosphoinositide phospholipase C (PLC, EC 3.1.4.11), 3′(2′),5′-bisphosphate nucleotidase 1 (SAL1, EC 3.1.3.57|3.1.3.7), inositol polyphosphate 5-phosphatase 9 (IP5P9, EC 3.1.3.36), inositol-phosphate phosphatase (IMPase, EC 3.1.3.25|3.1.3.93|3.1.3.15), myo-inositol-3-phosphate synthase (MIPS, EC 5.5.1.4), and methylmalonate-semialdehyde dehydrogenase (MMSDH, EC 1.2.1.27|1.2.1.18) (Figure 5A). Notably, the up-regulation of inositol-phosphate phosphatase, responsible for synthesizing inositol (the central metabolite of the analyzed pathway), is particularly significant.

In the case of RFO, it was noteworthy that all examined enzymes were UR (Figure 5B), spanning from galactinol synthase (GoIS, EC 2.4.1.123)—the enzyme linking the INS metabolic pathway with the RFO pathway—to the enzymes involved in synthesizing RFO itself, including raffinose synthase (RS, EC 2.4.1.82), stachyose synthase (STS, EC 2.4.1.67), invertase (or beta-fructofuranosidase) (INV, EC 3.2.1.26), and α-galactosidase (α-gal, EC 3.2.1.22).

**Figure 6:**
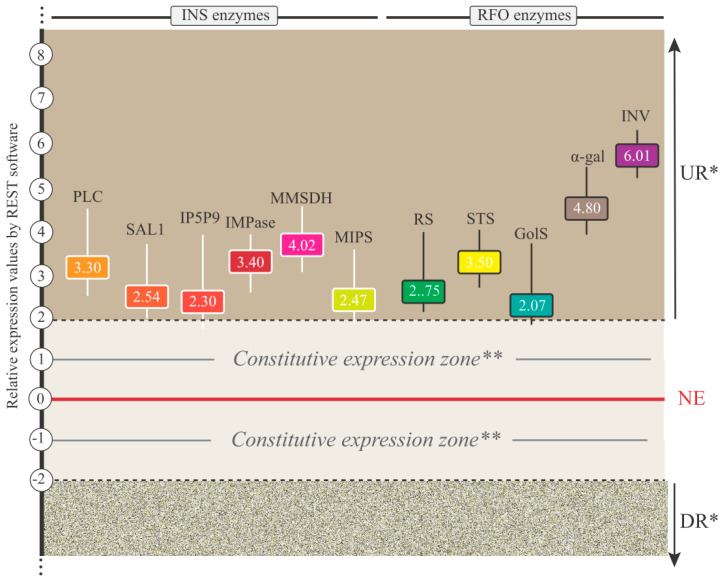
Relative expression values (numbers in rectangles) of 11 transcripts encoding various enzymes of the INS and RFO pathways, which exhibited up-regulation at 24 h water deprivation (WD.24h) treatment. The plotted values, along with the standard deviations of randomization tests represented by longitudinal lines extending from the rectangles, were derived from analyses performed using the REST software (version REST-2008). Legend: * −2 > relative expression > 2 and *p*-value < 0.05; ** −2 < relative expression < 2 and *p*-value > 0.05; UR: up-regulated; DR: down-regulated; NE: no expression; PLC: phosphoinositide phospholipase C, EC 3.1.4.11; SAL1: 3′(2′),5′-bisphosphate nucleotidase 1, EC 3.1.3.57|3.1.3.7; IP5P9: inositol polyphosphate 5-phosphatase 9, EC 3.1.3.36; IMPase: inositol-phosphate phosphatase, EC 3.1.3.25|3.1.3.93|3.1.3.15; MMSDH: methylmalonate-semialdehyde dehydrogenase, EC 1.2.1.27|1.2.1.18; MIPS: myo-inositol-3-phosphate synthase, EC 5.5.1.4; RS: raffinose synthase, EC 2.4.1.82; STS: stachyose synthase, EC 2.4.1.67; GoIS: galactinol synthase, EC 2.4.1.123; α-gal: α-galactosidase, EC 3.2.1.22; INV: invertase (or beta-fructofuranosidase), EC 3.2.1.26.

Additionally, the RNA-Seq data were validated through qPCR analyses. The expression levels of all 11 enzymes (from INS and RFO pathways) exhibiting UR were assessed. The results of the qPCR analyses confirmed the expression of all target transcripts, thereby underscoring the reliability of our transcriptomic analyses (Figure 6).

## 4. Discussion

### 4.1. INS and RFO Pathways in S. scabra: General Importance and Genomic Content Aspects

Genomically, identifying enzymes associated with the synthesis of INS and RFO in *S. scabra* (an important forage legume) is crucial for laying the groundwork for future biotechnological interventions, whether for gene overexpression or silencing. The biotechnological manipulation of these compounds is essential because they exert contrasting physiological effects depending on the target organism [29]. For mammals consuming them (*S. scabra* is an important forage), some INSs (e.g., phytate) [12] and RFO (e.g., raffinose and stachyose) [30] are considered antinutritional factors. Many breeding programs for legumes focus on genetically reducing the content of these antinutritional factors to ensure they are safe for consumption, thereby enhancing the utilization of grain legumes in animal diets. RFO, however, has recently been recommended in human diets to prevent cancer in the digestive tract [31]. In the context of plants, INS and RFO are believed to play crucial roles in various aspects of growth and adaptation [32].

Compared to other legumes, such as soybean (17 enzymes; [14]) and cowpea (21 enzymes; [15]), it was observed that *S. scabra* (and consequently, *A. hypogea*) presents actually the highest quantity of enzymes (22) genomically identified and associated with the INS pathway. From our data, it was found that out of the 24 compounds synthesized by the enzymes in question, 21 are different types of INS. Only enzymes synthesizing some of the stereoisomers of inositol or methylated derivatives (Figure 1A) were absent in all the legumes mentioned. Regarding the methylated derivatives, this lack of information likely stems from the fact that there has not yet been isolation and sequencing of genes associated with their synthesizing enzymes. Such compounds are present in the clade in question, being quantified in several representatives [33].

For the RFO pathway (Figure 1B), all evaluated enzymes were genomically identified in *S. scabra*. This observation keeps the legume group as a reference in the omics studies of this pathway. In addition to the enzymes synthesizing RFO themselves, the identification of galactinol synthase (GolS; EC 2.4.1.123) stood out, as it is the precursor of the mentioned pathway and is responsible for its connection with the INS metabolism.

Comparing *S. scabra* with soybean, another tetraploid legume with omics data available for the analyzed pathways reveals interesting differences. *S. scabra* exhibits higher values in terms of average loci number per enzyme compared to soybean—with 10.6 for INS and 14.5 for RFO, while soybean had 7.1 for INS and 11.0 for RFO [14]. This variation can be attributed to a combination of evolutionary factors, such as a genetic divergence between the two species and factors related to soybean domestication, including artificial selection and the reduction in natural selective pressure. Moreover, the difference may also reflect specific adaptations of metabolic pathways in each plant species to their respective environmental conditions. The presence of multiple genes for a given enzyme provides greater genetic flexibility, allowing species to adapt to extreme and constantly changing environments, such as the Caatinga semiarid region. In this environmental context, the greater quantity of INS and RFO loci in *S. scabra* becomes particularly significant.

### 4.2. Phylogenomic Dynamics of INS and RFO Synthesizing Gene Families

In addition to the genomic redundancy mentioned above, analysis using CAFE5 software (version 5) revealed that enzymes of the INS and RFO pathways are predominantly encoded by more than one gene family (Figure 2A,B). Interestingly, the majority of identified gene families in both pathways consist of one to four genes (Figure 2A,B). Gene families, as defined by OrthoFinder software (version 2.4.0), encompass homologous genes descended from a single gene of the last common ancestor of the examined species [16]. These copies can evolve independently, accumulating mutations over time, leading to sequence divergence, and potentially resulting in new functions or distinct transcriptional regulation, thus diversifying the molecular impact of the pathways under study.

Regarding the phylogenomic dynamics of gene family expansion or contraction in *S. scabra*, the phosphatidylinositol 4-phosphate 5-kinase 4-like (PIP5K; EC 2.7.1.68; Figure 2A) in the INS pathway exhibited a significant expansion (*p* < 0.05) of two gene families, while GolS (EC 2.4.1.123; Figure 2B) in the RFO pathway showed an expansion (*p* < 0.05) of one gene family. The expansion of these gene families reflects natural selection processes, suggesting their importance in the adaptation of *S. scabra* to the extreme Caatinga environment. PIP5K produces phosphatidylinositol (4,5)-biphosphate, a critical signaling phospholipid for various cellular processes in eukaryotes. According to Kuroda et al. [34], this enzyme has 11 genes in *A. thaliana*, and such entities play important roles in the physiology of this plant under stress. These authors observed that AtPIP5K7, AtPIP5K8, and AtPIP5K9 (orthologs to members of one of the expanded gene families in *S. scabra*) are not necessary under normal growth conditions but are redundantly involved in root growth adaptation to hyperosmotic conditions (i.e., dehydration-inducing conditions), possibly through the function of phosphatidylinositol (4,5)-biphosphate, promoting plasma membrane recycling in root meristem cells.

Regarding GolS, this enzyme is strategic because, as mentioned in the previous section, it connects the INS and RFO pathways. Overexpression of AtGolS2 (*A. thaliana* GolS 2) or PtrGolS3 (*Populus trichocarpa* GolS 3) in transgenic poplar (*Populus nigra*) resulted in improved performance and higher levels of soluble sugars and other metabolites (proline, salicylic acid, phenylalanine, etc.) in the genetically modified organism under salt stress (NaCl of 200 mM) compared to the wild-type control under the same condition [35].

### 4.3. Transcription Factors with Potential Regulatory Roles in the INS and RFO Pathways in S. scabra

Three bona fide CCRE, representing approximately 93% of the total identified, exhibited an average of at least one motif per promoter region investigated (Figure 3A,B). These regulatory elements showed an association with the transcription factors BCP1, DOF-type, and C_2_H_2_ (Figure 3B). This information underscores the significance of these transcription factors in potentially regulating the INS and RFO pathways in *S. scabra*, highlighting their biotechnological potential in this species.

Regarding BCP1 (BRCT5 DOMAIN CONTAINING PROTEIN 1), limited information exists regarding its impact on plants. Proteins containing the BRCT5 domain often play a role in preserving genome stability across eukaryotic organisms. Recently, a study identified BCP1 as a novel plant-specific DNA repair factor that acts in homology-based repair [36]. The enrichment of its presence in the promoter regions of genes associated with the INS and RFO pathways represents one of the initial indications of its association with specific metabolic pathways. The BBR/BPC family (to which BPC1 belongs) also performs several other functions in plant cells, such as participation in hormonal signaling processes, response to stresses, circadian rhythm regulation, and sexual determination [37].

Regarding DOF (DNA BINDING WITH ONE FINGER), these represent a family of transcription factors exclusively found in plants. Interestingly, in terms of comparative genomics, DOF is the only group mentioned above also enriched in promoters of the INS and RFO pathways in soybean [14] and cowpea [15], suggesting a significant association of these molecular actors with the INS and RFO pathways in legumes. DOF has been reported to play pivotal roles throughout the plant life cycle. They are involved in processes ranging from the regulation of hormonal signals and developmental processes (such as dormancy, tissue differentiation, among others) to responses to biotic or abiotic stresses [38].

C_2_H_2_, in turn, is considered master regulators of responses to abiotic stresses commonly found in Caatinga, such as high luminosity, high salinity, drought, and osmotic disturbances [39]. Their association with the INS and RFO pathways in *S. scabra* underscores their importance in regulating molecular mechanisms with a significant influence on plant physiology.

Lastly, it is worth highlighting that our previous analyses have demonstrated that DOF and C_2_H_2_ are among the top ten transcription factor families with the highest number of up-regulated representatives in *S. scabra* under water deprivation [2]. This suggests the significance of these molecular actors—and their downstream regulatory targets, including protein-coding genes and metabolic pathways—under the mentioned stressful condition.

### 4.4. In Silico Prediction of Potential Functions of the INS and RFO Pathways

The group of terms exhibiting a specific action is diverse, encompassing several terms (highlighted in red rectangles in Figure 4) associated with adaptive responses to conditions commonly encountered in extreme environments. This diverse array of strategic functions, potentially involving the analyzed pathways, underscores their significance for *S. scabra*.

A pivotal finding from this analysis is the responsiveness of the INS and RFO pathways to the phytohormone abscisic acid (ABA) (Figure 4). ABA is indispensable for plant growth and development, acting as a crucial regulator in integrating various stress signals and coordinating downstream stress responses [40]. Plants must continually adjust ABA levels in reaction to changing physiological and environmental conditions [40].

Furthermore, the other enriched terms intriguingly mirror the natural conditions of the Caatinga semiarid environment, i.e., water deprivation, osmotic stress, salt stress, and desiccation. Numerous scientific studies have demonstrated the involvement of INS and RFO in responding to such conditions. Regarding the INS pathway, the study by Li et al. [41] is notable. These authors suggested that inositol-induced drought tolerance contributed to better water relations by enhancing osmotic adjustment and water use efficiency, reducing chlorophyll loss for photosynthetic maintenance, and enhancing antioxidant enzyme activity and gene expression, thereby mitigating oxidative damage under drought stress.

As for the RFO pathway, it was observed that exposing 5-day-old pea seedlings to osmotic stress (induced by immersing roots in a PEG8000 solution at −1.5 MPa) for 48 h resulted in the synthesis of galactinol and RFO (raffinose and stachyose) in both epicotyl and root tissues [42]. After 24 h of recovery, galactinol completely disappeared, while raffinose decreased fourfold and stachyose decreased twofold in roots [42]. Additionally, Nishizawa-Yokoi et al. [43] proposed that carbohydrates, including RFO, are present at high levels under normal and/or stressful conditions and act as antioxidants, safeguarding plant cells against oxidative damage and maintaining redox homeostasis.

### 4.5. The S. scabra Transcriptional Orchestration of INS and RFO Pathways under Water Deprivation

The transcriptional orchestration of the INS and RFO pathways during the studied stressful condition underscores their significance in the molecular physiology of *S. scabra*. Remarkably, about 87% of the INS- and 100% of the RFO-enzymes displayed no transcriptional bottlenecks, either being UR or constitutively expressed (Figure 5A,B). Notably, all 11 UR enzymes (for INS and RFO pathways) were validated for their expression by qPCR assay (Figure 6), consolidating their association with mechanisms of acclimation to dehydration stressors in plants. These findings enhance our understanding of the functional potential of these enzymes under stress conditions.

#### 4.5.1. UR INS-Enzymes in *S. scabra* Response to Water Deprivation

Phosphoinositide phospholipase C (PLC; EC 3.1.4.11) (Figure 5A and Figure 6) responds to adverse external conditions by generating 1,2-Diacylglycerol and 1D-myo-Inositol 1,4,5-trisphosphate, a significant secondary messenger [44]. Recent studies have demonstrated that overexpression of AtPLC3 and AtPLC5 in *A. thaliana* reduced stomatal aperture, leading to decreased water loss through transpiration and enhanced drought tolerance [45,46]. Conversely, overexpression of AtPLC7 also increased drought tolerance but did not affect stomatal aperture [47].

Another enzyme, methylmalonate-semialdehyde dehydrogenase (MMSD; EC 1.2.1.27|1.2.1.18) (Figure 5A and Figure 6), links the INS pathway to the tricarboxylic acid cycle. Up-regulation of MMSD has been reported in drought-tolerant cowpea accessions, suggesting its role in response to root dehydration [15]. While its functional characterization under abiotic stress conditions remains unexplored, previous studies have implicated MMSD in root formation in rice plants [48], a critical response to drought conditions.

Inositol-phosphate phosphatase (IMPase; EC 3.1.3.25|3.1.3.93|3.1.3.15) (Figure 5A and Figure 6) catalyzes the final step of inositol biosynthesis. Their overexpression in transgenics has been associated with better performance of genetically modified organisms when subjected to osmotic perturbations. Wang et al. [49] observed that the deletion of the all2917 gene (encoding an IMPase) in *Anabaena* sp. PCC7120 (a filamentous cyanobacterium) made it more sensitive to osmotic stress caused by sucrose or sorbitol. Conversely, its overexpression resulted in increased tolerance to such stress. Consistent with the above observations, the transcription of all2917 was significantly induced in Anabaena sp. PCC7120 under sucrose-mediated osmotic stress conditions.

Das-Chatterjee et al. [50], in turn, demonstrated that the functional introgression of the PcINO1 gene, which encodes a myo-inositol-3-phosphate synthase (MIPS; EC: 5.5.1.4) (Figure 5A and Figure 6) in *Porteresia coarctata*, conferred varying degrees of salt stress tolerance (NaCl) in evolutionarily diverged organisms, spanning from prokaryotes to eukaryotes, including cultivated plants. Their study revealed a direct correlation between the enhanced synthesis of inositol under salt stress mediated by the PcINO1 enzyme and the observed salt tolerance levels.

Regarding the SAL1 enzyme (EC 3.1.3.57|3.1.3.7) (Figure 5A and Figure 6), it is well-established in the scientific literature that the sustained high-level accumulation of the chloroplast stress signal, 3′-phosphoadenosine-5′-phosphate (PAP), confers drought tolerance to plants while impeding plant growth and development [51]. PAP, a by-product of sulfur metabolism, is typically maintained at minimal levels by SAL1 during the vegetative growth of Arabidopsis but accumulates in rosettes during drought and excessive light conditions [51]. In *S. scabra*, SAL1 expression was observed up-regulated in response to WD.24h treatment, indicating species-specific functionality under stress and the ability of *S. scabra* to sustain low PAP levels, even in adverse conditions.

Finally, concerning the INS pathway, we observed the up-regulation of inositol polyphosphate 5-phosphatase 9 (IP5P9; EC 3.1.3.36) (Figure 5A and Figure 6), which currently lacks functional characterization data in any organism. Regarding its transcriptomics, our group reported its constitutive expression in cowpea under root dehydration for up to 150 min [15]. This data, combined with the information presented here, suggests that this enzyme displays species-specific expression patterns in legumes.

#### 4.5.2. UR RFO-Enzymes in *S. scabra* Response to Water Deprivation

All studied RFO-enzymes displayed elevated expression levels in response to the analyzed stress (Figure 5B and Figure 6). The concurrent up-regulation of these molecular actors suggests coordinated redirection of metabolic flux to meet the heightened demand for stress-related metabolites. Furthermore, the up-regulation of entire metabolic pathways underscores the intricate nature of the *S. scabra* stress responses. It emphasizes the multifaceted aspect of stress adaptation, which involves not only individual genes but also entire biochemical routes.

Galactinol synthase (GoIS, EC 2.4.1.123), the pivotal enzyme for connecting the INS and RFO metabolisms (Figure 5B), has been linked to improving the resilience of genetically modified organisms under challenging environmental conditions. In a study conducted by Vinson et al. [52], transgenic Arabidopsis plants overexpressing AdGolS3 (*Arachis duranensis* GolS3) showed increased levels of raffinose. They demonstrated decreased stress symptoms when subjected to drought, osmotic, and salt stresses. Analysis of metabolites and gene expression patterns indicated a correlation between AdGolS3 overexpression and mitigated metabolic disruptions under drought stress, as well as enhanced protection against oxidative stress [52].

Raffinose synthase (RS, EC 2.4.1.82) is responsible for synthesizing raffinose by transferring a galactosyl group from galactinol to sucrose (Figure 5B). Li et al. [10] observed that mutant RS maize plants lacking raffinose and accumulating galactinol displayed heightened sensitivity to drought stress compared to null-segregant plants. Conversely, overexpression of ZmRS (*Zea mays* Raffinose Synthase) in *A. thaliana* improved the tolerance to drought stress [10].

Plant invertases (INV, EC 3.2.1.26) (Figure 5B and Figure 6) are key players in directing carbon allocation from source tissues (such as autotrophic leaves) to storage organs like seeds, tubers, and fruits. Moreover, invertases contribute significantly to plant growth and responses to both biotic and abiotic stresses [53]. A comprehensive analysis conducted by Abbas et al. [54] shed light on the gene family of INV, revealing distinct expression patterns across various tissues of potato plants. While INV genes exhibited higher expression in flowers, leaves, roots, and shoots, the expression levels of some of them were notably up-regulated under abiotic stress conditions (salt-150 mM NaCl, mannitol-250 µM, BAP-10 µM, ABA 50 µM, IAA-10 µM, GA3-50 µM, heat 35 °C for 24 h, primary and secondary tissue wounding, and BABA and BTH treatment for 24 h/48 h/72 h).

Lastly, the functional consequences of up-regulating stachyose synthase (STS, EC 2.4.1.67) and α-galactosidase (α-gal, EC 3.2.1.22) (Figure 5B and Figure 6) enzymes in plants under stress conditions remain to be fully understood. Considering the comparative legume transcriptomics, our group also noted the up-regulation of α-gal in cowpea under treatments of up to 150 min of root dehydration [15]. This observation highlights its significance as a potential candidate for subsequent functional analyses.

## 5. Conclusions

The INS and RFO pathways play pivotal roles in influencing plant physiology, particularly under stressful conditions, where they serve as vital secondary messengers, antioxidants, and other important functions. Our assessment of these metabolic routes in *S. scabra* (a species native to the harsh Caatinga environment) through omics approaches contributes significantly to our understanding of both the species and the molecular mechanisms at play. Among legumes, *S. scabra* (along with *A. hypogea*) stood out for the abundance of identified enzymes from these pathways. Notably, these enzymes exhibited genomic redundancy, being encoded by multiple loci and various gene families. The phylogenomic analysis revealed no expansion or contraction of these genes relative to their immediate *S. scabra* ancestor, except for PIP5K and GolS, which are crucial in synthesizing, respectively, key secondary messengers and the RFO precursor. Transcriptional control of the referred pathways appears to be closely associated with DOF-type, C_2_H_2_, and BCP1 transcription factors. While DOF-type and C_2_H_2_ are commonly linked to stress responses, BCP1’s role in plants remains less understood, warranting further investigation. The analysis of biological processes related to INS and RFO pathways in *S. scabra* highlights their significance in responding to stressful conditions prevalent in the Caatinga. Finally, transcriptomic data (RNA-Seq and qPCR) revealed the extent of influence and activity of the INS and RFO pathways in *S. scabra* under water deprivation: about 87% of the INS pathway showed no transcriptional bottleneck (i.e., were up-regulated or constitutively expressed) under the analyzed stress; for RFO, it was found that 100% of the evaluated enzymes were up-regulated. Especially for the INS pathway, the scrutinized treatment time (WD.24h), an initial stress exposure time considering the applied methodology, may be the driving force behind this result since the pathway in question is a crucial source of important secondary messengers. Our study deciphers the genetics and transcriptomics of the INS and RFO pathways in *S. scabra*, shedding light on their importance for a Caatinga plant and paving the way for future biotechnological applications in this species and beyond.

## Data Availability

The genomic and RNA-Seq raw reads substantiating the findings of this article are accessible in the NCBI Bioproject repository (https://www.ncbi.nlm.nih.gov/bioproject/, under the accession PRJNA924790, and in the NCBI SRA (https://www.ncbi.nlm.nih.gov/sra) repository, under the accession PRJNA837909, respectively.

## Supplementary Materials

The following supporting information can be downloaded at: https://www.mdpi.com/article/10.3390/plants13131749/s1. Supplementary Material S1. Melting curves for target transcripts analyzed in the present work. Supplementary Material S2. Reference genes and target transcripts utilized in the qPCR relative expression analysis, detailing their annotations, primer pair sequences, RNA-Seq modulation, and primer pair efficiency values.
