# Supplementary material for: From Genes to Stress Response: Genomic and Transcriptomic Data Suggest the Significance of the Inositol and Raffinose Family Oligosaccharide Pathways in Stylosanthes scabra, Adaptation to the Caatinga Environment
Source: Plants (Basel). 2024 Jun 25;13(13):1749. doi: 10.3390/plants13131749 (PMC11243744; doi:10.3390/plants13131749)

MELTING CURVES

Ssc_TR29992|c1_g1_i1

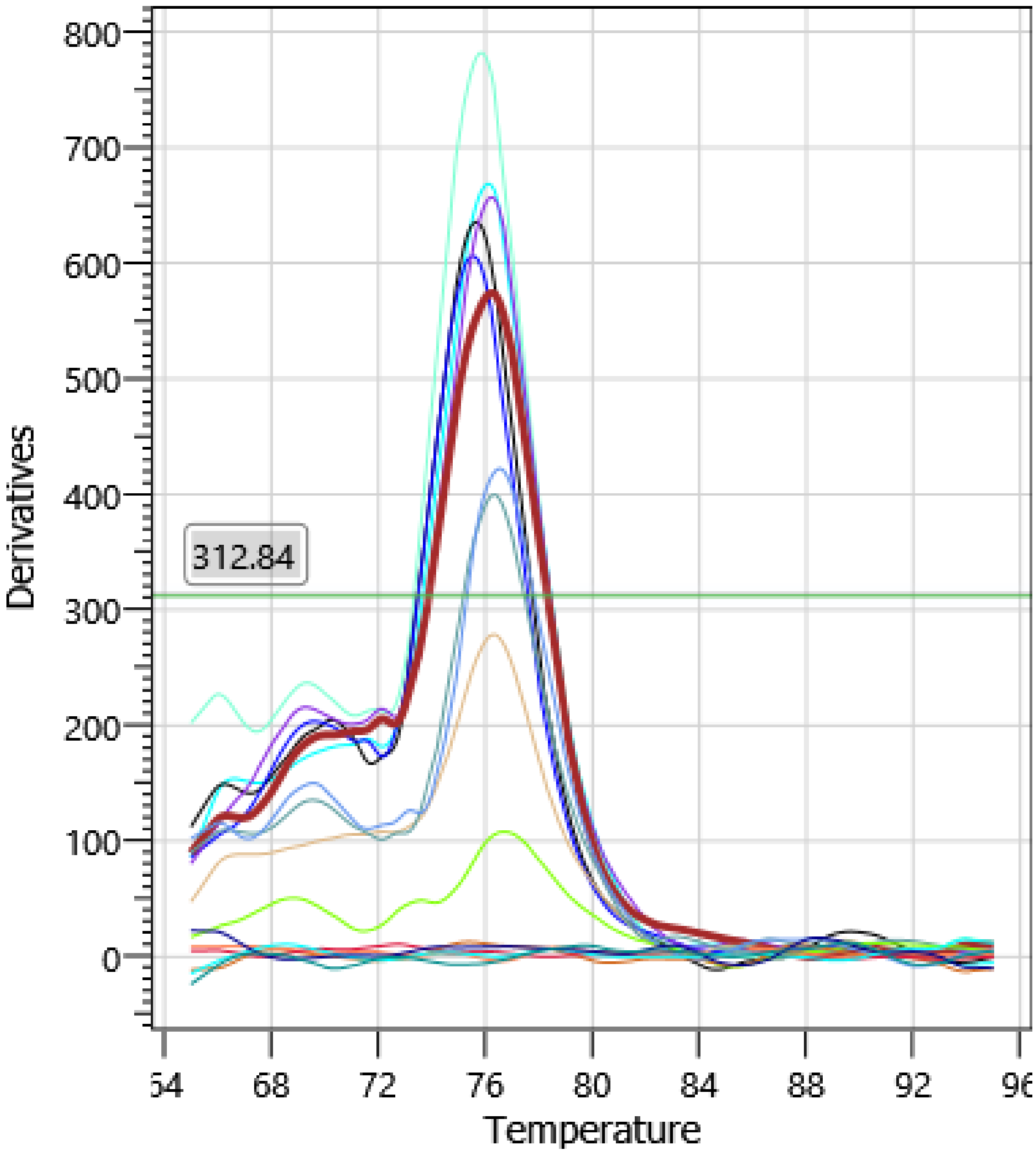

Ssc_TR68754|c1_g1_i2

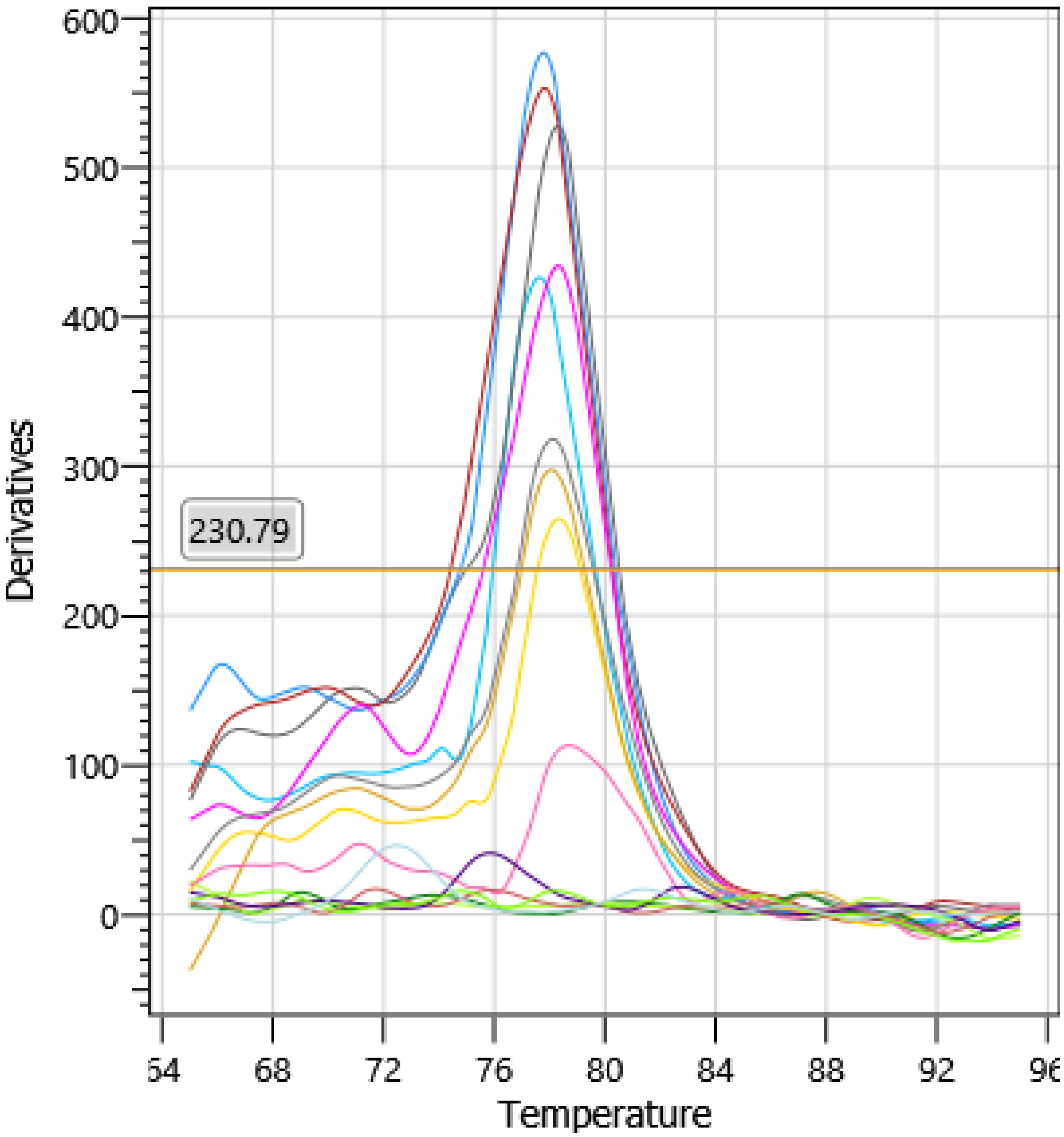

Ssc_TR25515|c1_g1_i1

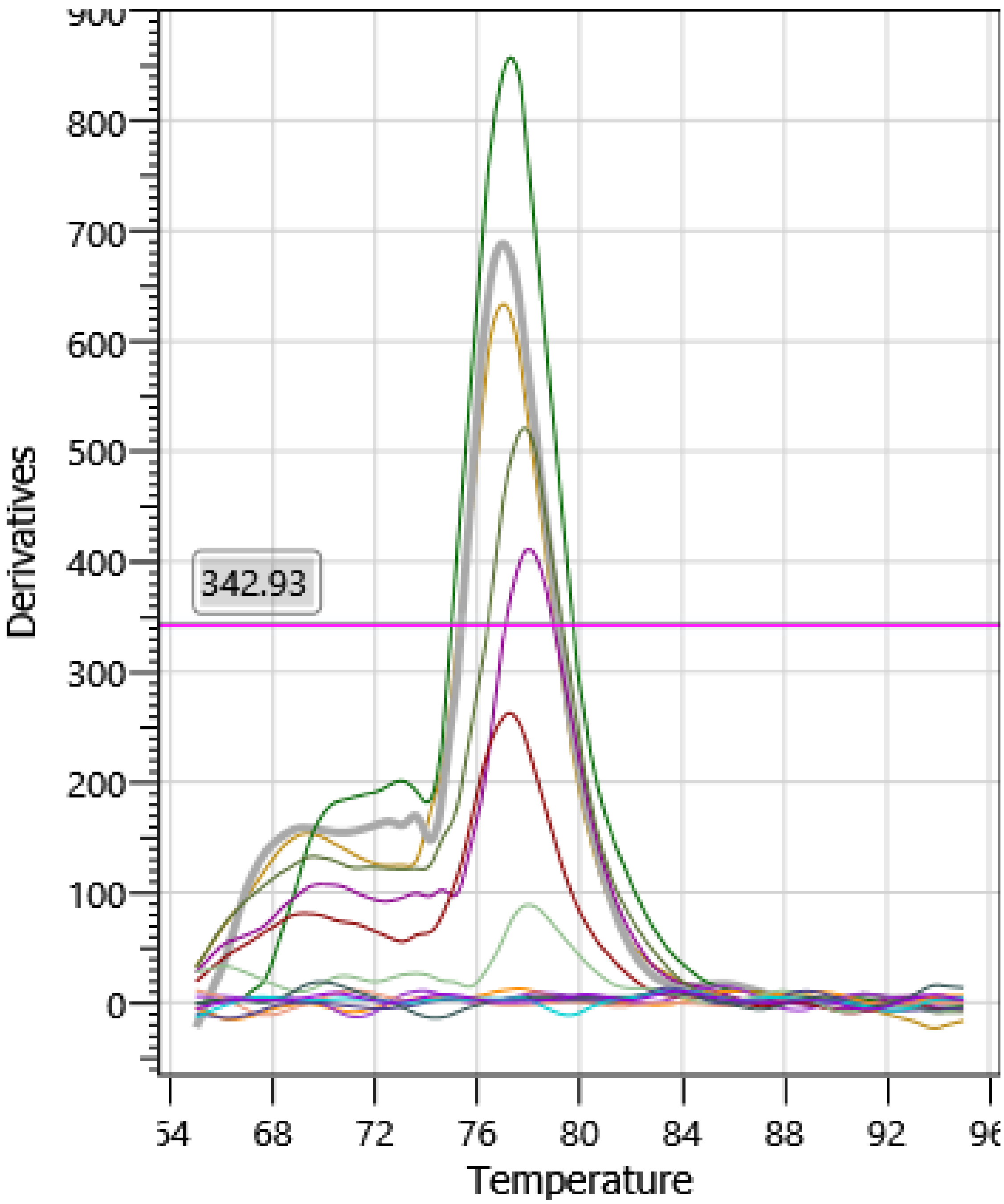

Ssc_TR30519|c1_g1_i10

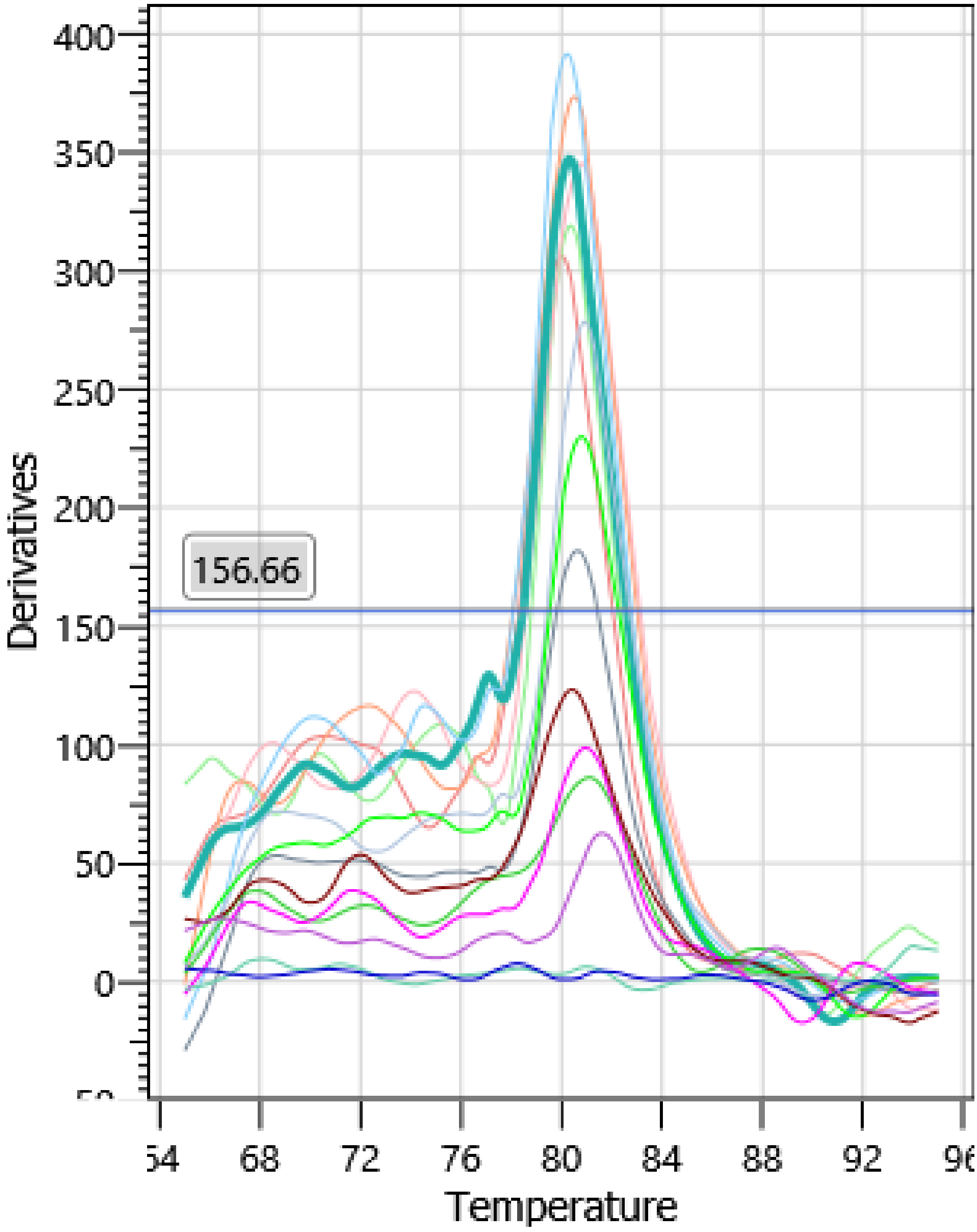

Ssc_TR28285|c1_g1_i3

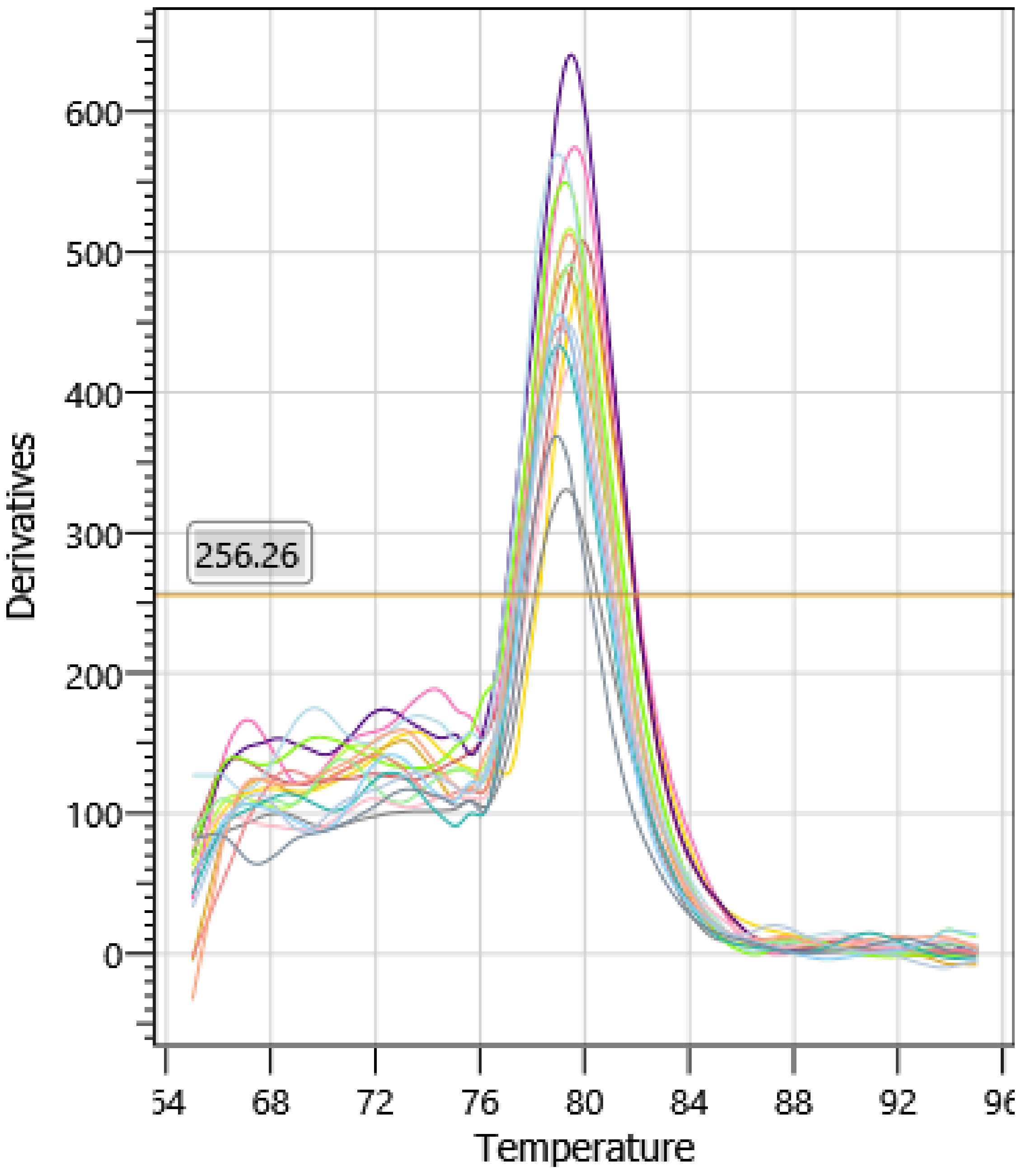

Ssc_TR69066|c2_g2_i1

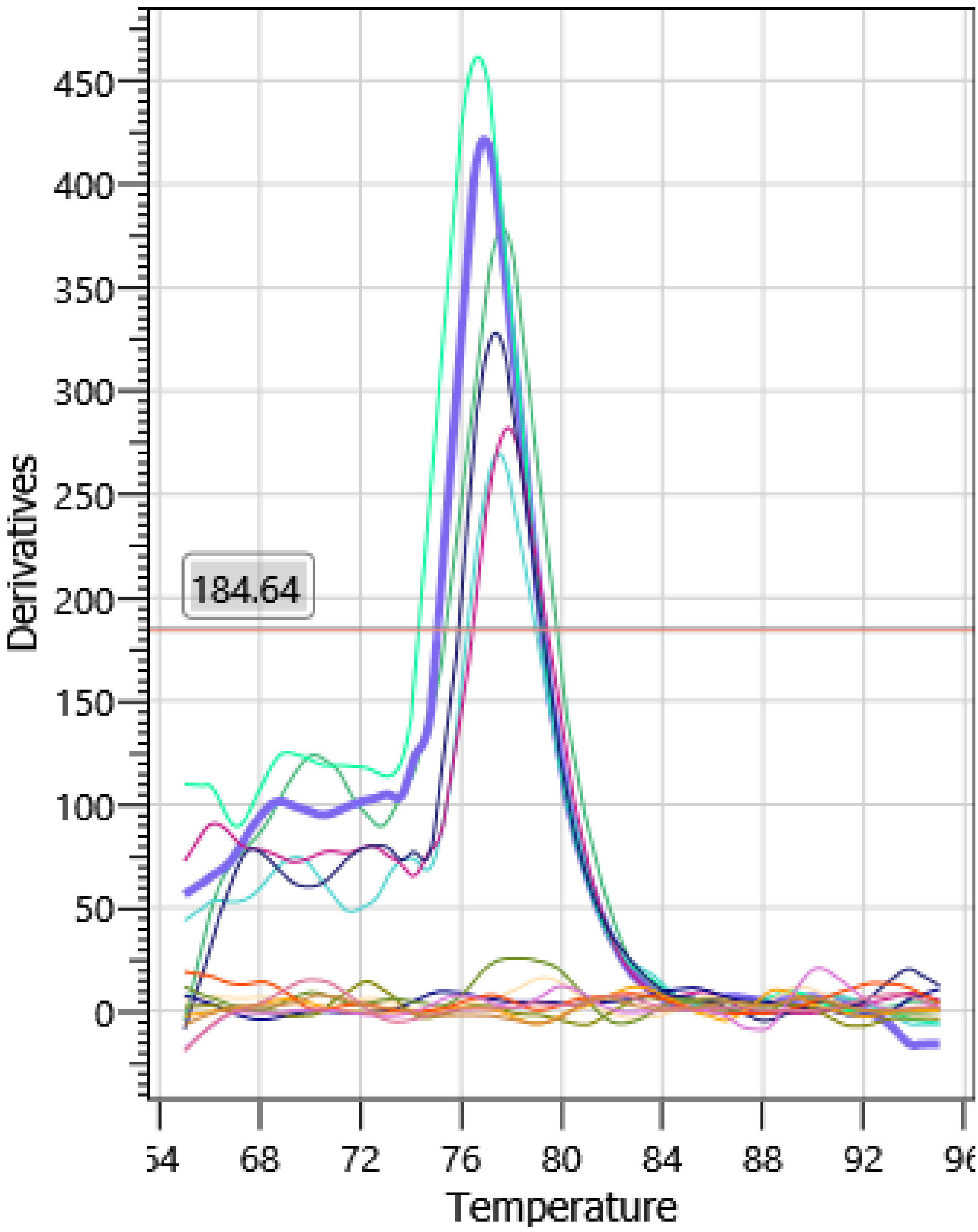

Ssc_TR29676|c2_g2_i2

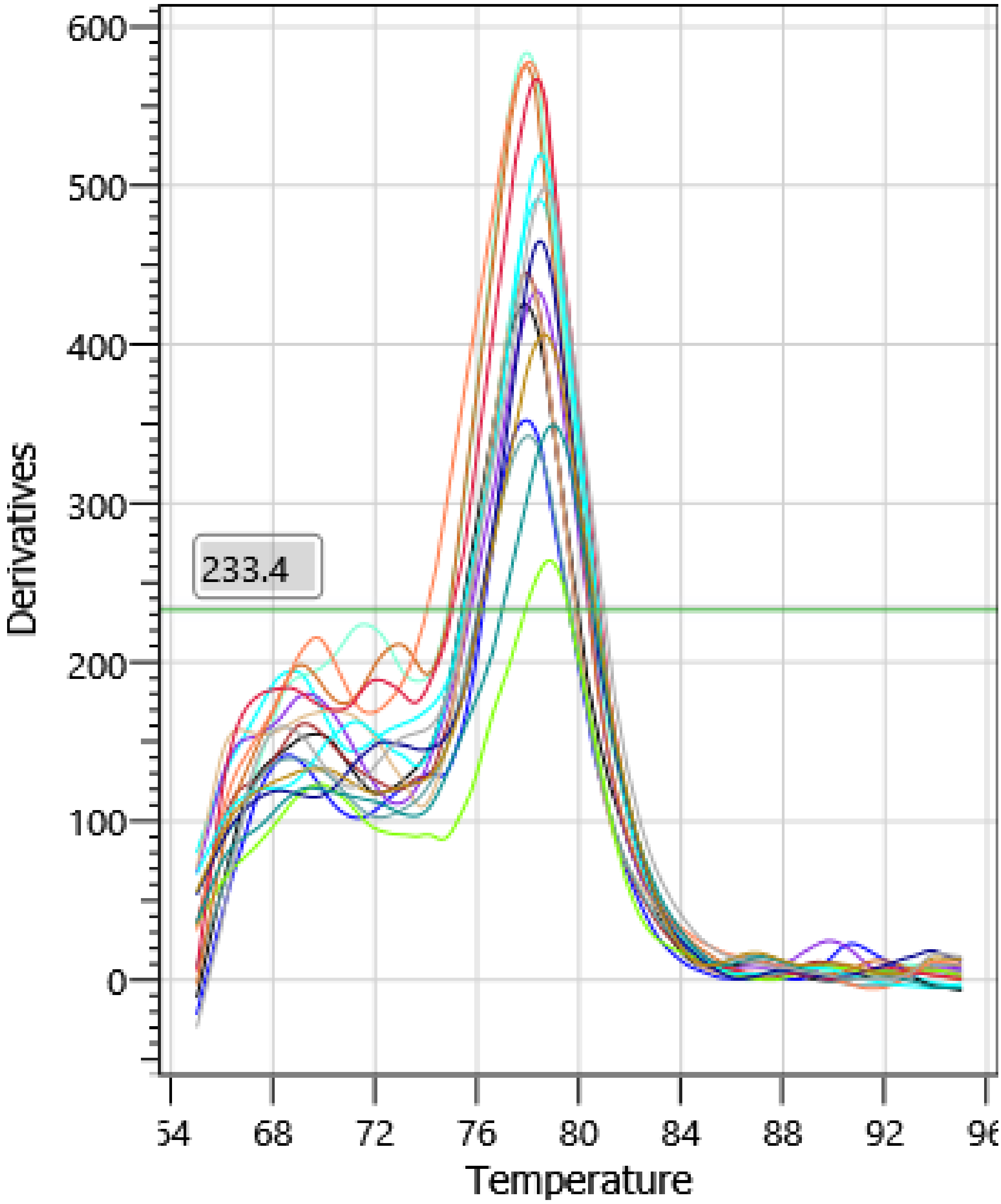

Ssc_TR69684|c0_g1_i6

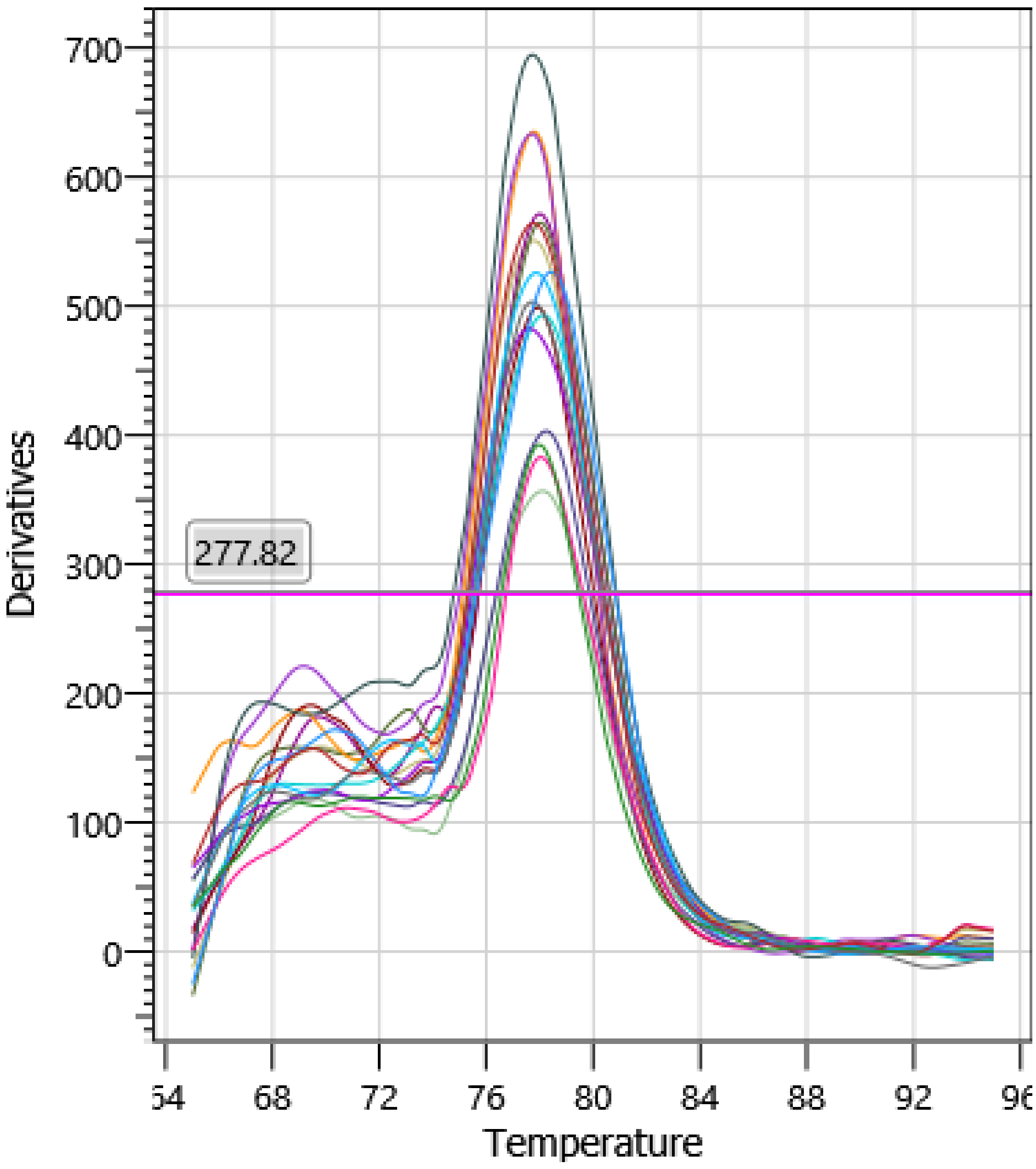

Ssc_TR68151|c0_g1_i2

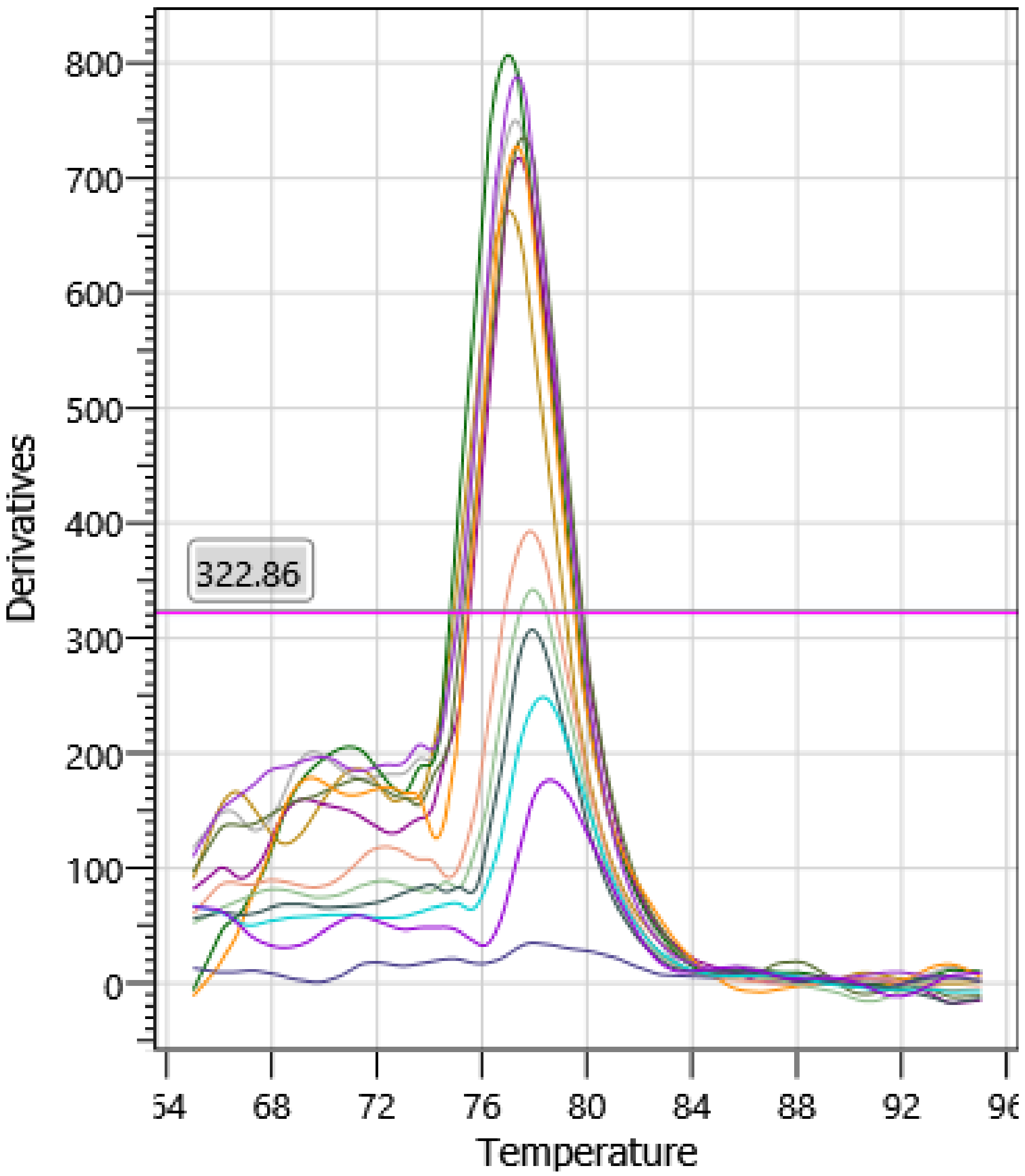

Ssc_TR25712|c0_g3_i2

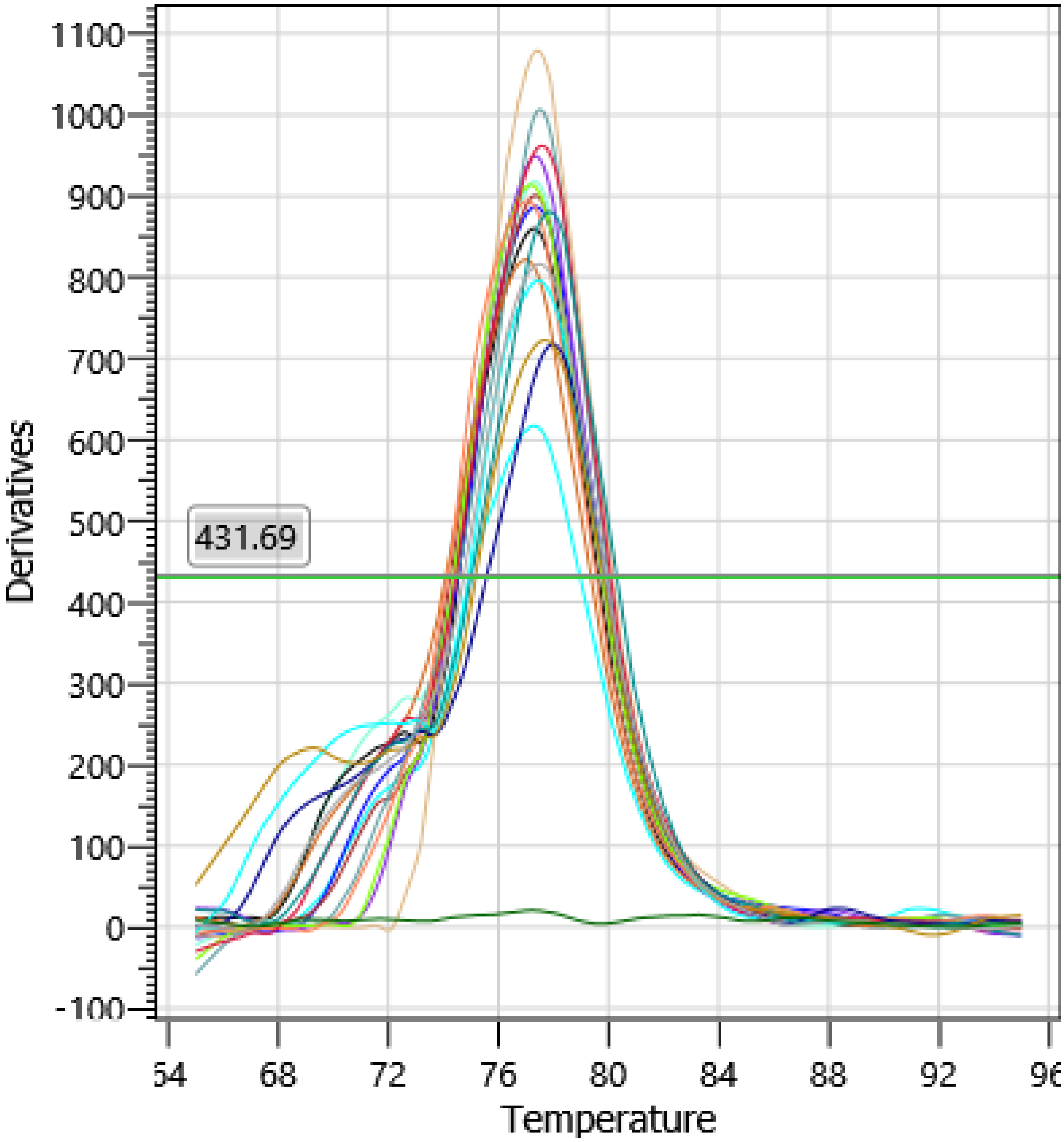

Ssc_TR29592|c0_g1_i3

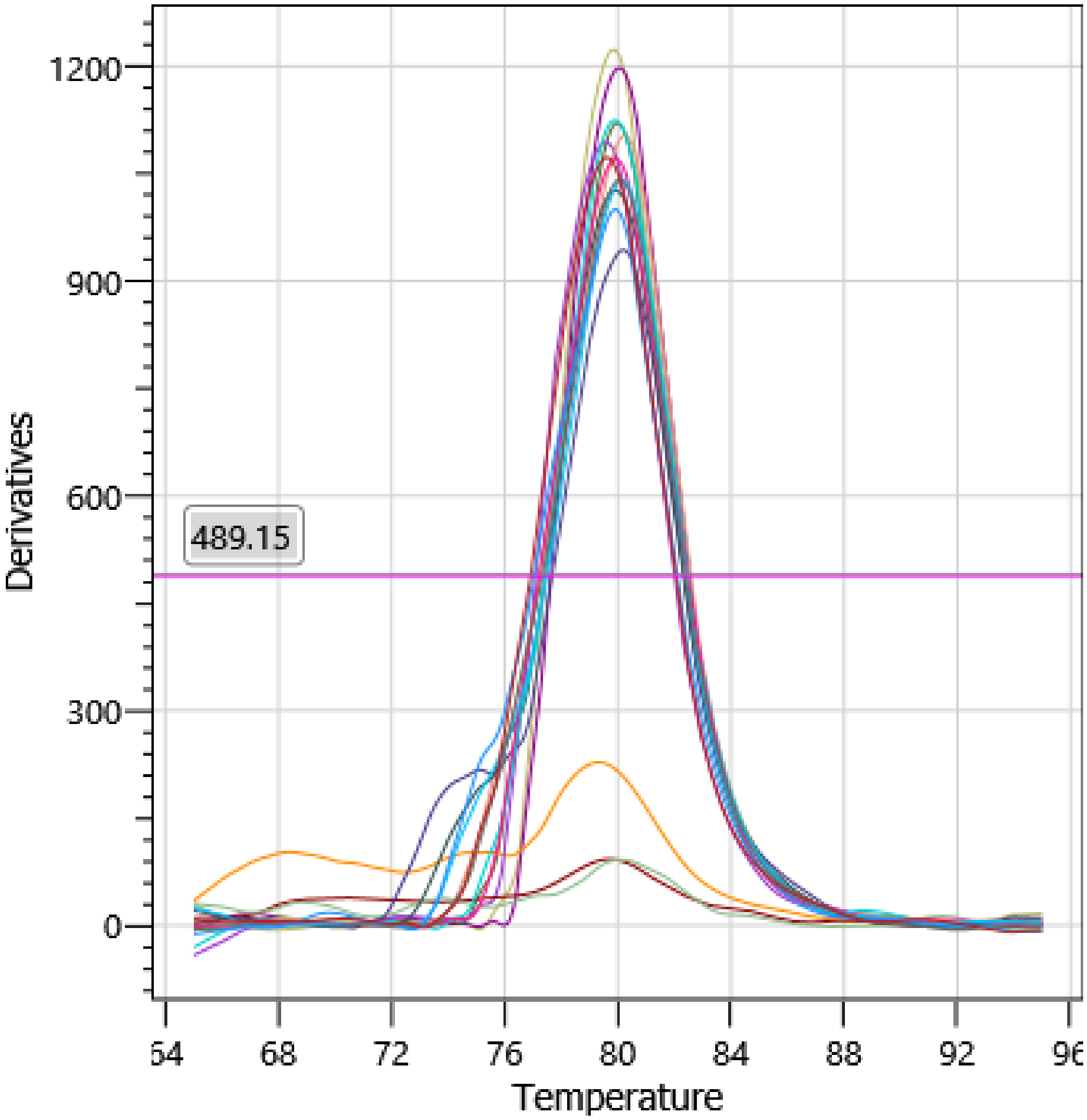

Supplement: Supplementary file 1 [file plants-13-01749-s001.zip › Supplementary Material S1.pdf]
